# Compounds targeting GPI biosynthesis or *N*-glycosylation are active against *Plasmodium falciparum*

**DOI:** 10.1016/j.csbj.2022.01.029

**Published:** 2022-02-02

**Authors:** Àngel Fenollar, Albert Ros-Lucas, María Pía Alberione, Nieves Martínez-Peinado, Miriam Ramírez, Miguel Ángel Rosales-Motos, Ling Y. Lee, Julio Alonso-Padilla, Luis Izquierdo

**Affiliations:** a Barcelona Institute for Global Health (ISGlobal), Hospital Clínic—University of Barcelona, 08036 Barcelona, Spain; b CIBER de Enfermedades Infecciosas, Madrid, Spain

**Keywords:** Malaria, *Plasmodium falciparum*, GPI-anchors, *N*-glycosylation, Inhibitors, Antiplasmodial activity, GlcNAc-PI de-*N*-acetylase, *N*-acetylglucosaminyl-phosphatidylinositol de-*N*-acetylase, GWT1, Inositol acyltransferase, GPI, Glycosylphosphatidylinositol, IDC, Intraerythrocytic developmental cycle, ALG7, UDP-*N*-acetylglucosamine:dolichyl-phosphate*N*-acetylglucosaminephosphotransferase, CSP, Circumsporozoite protein, ER, Endoplasmic reticulum, *N*-glycans, Asparagine-linked glycans, OST, Oligosaccharyltransferase, GlcNAc, *N*-acetylglucosamine, CDS, Coding sequence, SHAM, Salicylic hydroxamic acid, pLDDT, Predicted local distance difference test, UDP-GlcNAc, UDP-*N*-acetylglucosamine, DMSO, Dimethyl sulfoxide, RBCs, Red blood cells, GSL-II, *Griffonia simplicifolia*II lectin

## Abstract

The emergence of resistance to first-line antimalarials, including artemisinin, the last effective malaria therapy in some regions, stresses the urgent need to develop new effective treatments against this disease. The identification and validation of metabolic pathways that could be targeted for drug development may strongly contribute to accelerate this process. In this study, we use fully characterized specific inhibitors targeting glycan biosynthetic pathways as research tools to analyze their effects on the growth of the malaria parasite *Plasmodium falciparum* and to validate these metabolic routes as feasible chemotherapeutic targets. Through docking simulations using models predicted by AlphaFold, we also shed new light into the modes of action of some of these inhibitors. Molecules inhibiting *N*-acetylglucosaminyl-phosphatidylinositol de-*N*-acetylase (GlcNAc-PI de-*N*-acetylase, PIGL/GPI12) or the inositol acyltransferase (GWT1), central for glycosylphosphatidylinositol (GPI) biosynthesis, halt the growth of intraerythrocytic asexual parasites during the trophozoite stages of the intraerythrocytic developmental cycle (IDC). Remarkably, the nucleoside antibiotic tunicamycin, which targets UDP-*N*-acetylglucosamine:dolichyl-phosphate *N*-acetylglucosaminephosphotransferase (ALG7) and *N*-glycosylation in other organisms, induces a delayed-death effect and inhibits parasite growth during the second IDC after treatment. Our data indicate that tunicamycin induces a specific inhibitory effect, hinting to a more substantial role of the *N*-glycosylation pathway in *P. falciparum* intraerythrocytic asexual stages than previously thought. To sum up, our results place GPI biosynthesis and *N*-glycosylation pathways as metabolic routes with potential to yield much-needed therapeutic targets against the parasite.

## Highlights

• Compounds targeting key steps in GPI biosynthesis abrogate *P. falciparum* growth.
• *N*-glycosylation disruption halts parasite development and induces delayed death.
• Tunicamycin-induced delayed death is not linked with the synthesis of isoprenoids.
• In summary, two metabolic pathways are outlined for further drug target exploration.

## Graphical abstract

**Figure:**
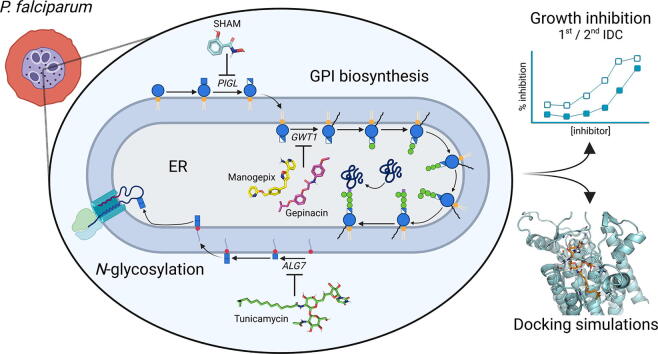

## 1 Introduction

Only in 2020, mosquito-transmitted malaria caused 241 million cases and killed more than 600,000 people, most of them children below five years in sub-Saharan Africa [1]. Despite calls for malaria elimination during the last decade, and the significant case and death reduction from the beginning of the 21st century, the disease still represents an unacceptable burden and the rate of decline of both cases and deaths has recently stalled [2]. Furthermore, the ability of *Plasmodium* spp., the parasite that causes the disease, to develop resistance to all of the currently available antimalarial drugs [3], [4], including artemisinin [5], highlights the urgent need to characterize new drug targets and to develop new antimalarial drugs, for both prophylaxis and chemotherapy.

Five *Plasmodium* species can cause malaria in humans: *P. falciparum*, *P. vivax*, *P. malariae*, *P. ovale*, and *P. knowlesi* [6]. *P. falciparum* and *P. vivax* are the responsible for most cases, although most severe complications and malaria deaths are caused by *P. falciparum* [1]. The infection starts with the inoculation of sporozoites into the skin of the host by an infected *Anopheles* mosquito. Sporozoites reach the bloodstream and travel through the blood vessels to the liver to invade hepatocytes and initiate the liver stage. Once mature, infected hepatocytes burst and release exoerythrocytic merozoites that invade erythrocytes, initiating the asexual blood stage of the infection responsible of malaria symptoms, in which the parasite goes through multiple rounds of intraerythrocytic replication. From the parasites that continue this 48 h long asexual cycle, a small fraction of them differentiate into intraerythrocytic male and female gametocytes (sexual forms), which can be taken up by mosquitoes, progress through the mosquito stages and, ultimately, infect new human hosts [7].

Glycoconjugates on the cell surface of protozoan parasites play key roles in determining parasite-host interactions and survival [8]. Glycosylation reactions are catalyzed by glycosyltransferases, which attach sugar moieties to glycoprotein or glycolipid acceptors using activated sugar nucleotide donors. Despite the existing gaps of knowledge [9], the malaria parasite does not seem to produce many complex glycoconjugates [10], [11], [12]. Nevertheless, several functional glycosyltransferases are conserved and expressed in the genome of *P. falciparum* [13], [14], [15], [16], and different sugar nucleotide precursors are detected in the asexual and sexual parasite blood stages [17], [18], [19].

The most prominent form of protein glycosylation in the malaria parasite is the addition of a glycosylphosphatidylinositol (GPI) to the C-terminus of certain proteins, to anchor them into lipid bilayers [20]. *P. falciparum* GPI-anchors consist of a lipid moiety attached to a conserved core composed of an acylated inositol ring, a glucosamine residue and 3/4 mannoses, linked to an ethanolamine [10]. The ethanolamine moiety is bound to the C-terminus of the anchored protein by an amide linkage. Various GPI-anchored proteins, including merozoite surface protein 1 (MSP-1) [21], Pfs48/45 [22] or circumsporozoite protein (CSP) [23], are essential for the development of *P. falciparum* along different stages of its life cycle. GPI-anchors, which act as a pro-inflammatory endotoxin in the infected host [24], are synthesized in the endoplasmic reticulum (ER) by the coordinated sequential action of several enzymes and enzymatic complexes [25]. These enzymatic steps are mainly hypothesized by the presence of orthologs in the *P. falciparum* genome, but their function and relevance for parasite development has not been experimentally confirmed [20], [26].

Most eukaryotic organisms, from yeast to mammals, add asparagine-linked glycans (*N*-glycans) to proteins expressed in the secretory pathway [27], [28]. This post-translational modification, essential in most eukaryotes, modulates folding, stability and protein function and trafficking [29]. *N*-glycosylation is a sequential process in which a lipid-linked glycan precursor is synthesized by specific glycosyltransferases, known as ALG (from Asparagine linked glycosylation), before being transferred to specific asparagine residues (N-X-T/S) on nascent proteins in the ER by the oligosaccharyltransferase (OST) protein complex [30]. Remarkably, while higher eukaryote *N*-glycan precursors contain 14 sugars synthesized by the sequential action of 12 enzymes, protists present different sets of ALG genes, affecting to the final composition of the glycan donor [31]. Likewise, besides the STT3 catalytic subunit of OST, the number of subunits composing this hetero oligomeric complex vary in different organisms [30]. In the case of *P. falciparum*, the parasite only presents ALG7, ALG13 and ALG14 glycosyltransferases, and makes lipid-linked glycan precursors containing one or two residues of *N*-acetylglucosamine (GlcNAc) [11]. Regarding the OST complex, seven subunits, including the STT3 catalytic subunit, have been identified in the genome of the parasite [32].

Considering the importance of GPI-anchors and *N*-glycosylation for most eukaryotes, in this work we have assessed the relevance of these poorly explored pathways for the survival of the malaria parasite by taking advantage of specific inhibitors as tools to probe these pathways. Some of these compounds have been recently described and/or are in clinical trials for the treatment of fungal infections [33], [34]. Our data strongly suggest that inhibiting GPI and *N*-glycosylation provokes parasite death in the asexual blood stages of parasite development. Hence, these biosynthetic routes deserve further exploration as potential new sources of much needed antimalarial drug targets.

## 2 Results

### 2.1 *P. falciparum* orthologs involved in GPI-anchor and *N*-glycan biosynthesis

**Fig. 1:**
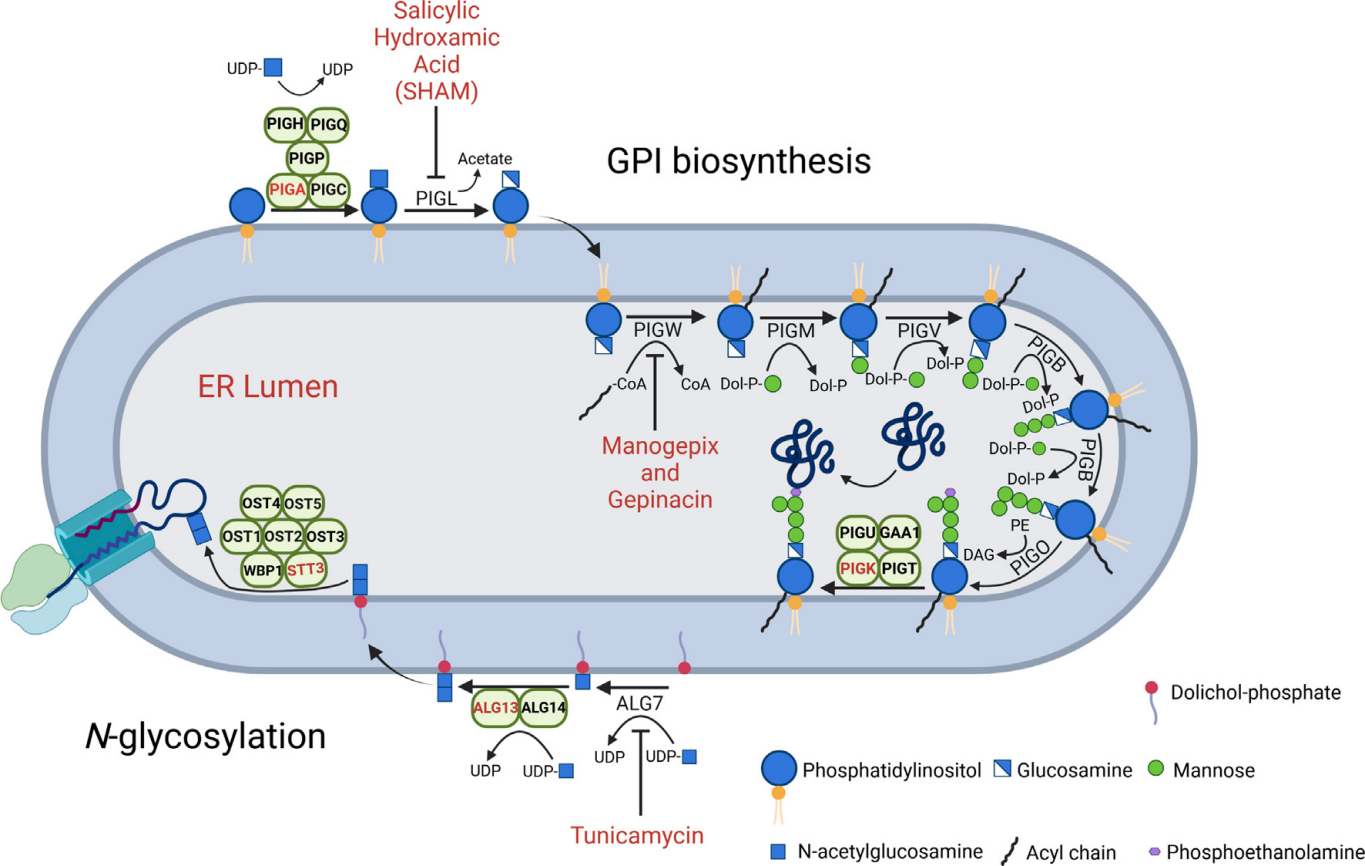
Biosynthesis of GPI anchors and *N*-glycosylation in the *P. falciparum* ER. Enzyme names are indicated in Table 1 and catalytic subunits are highlighted in red. The different compounds tested in this work and the enzymatic steps they inhibit are also displayed. The predicted byproducts of every reaction are included in the illustration. (For interpretation of the references to colour in this figure legend, the reader is referred to the web version of this article.)

A survey of the *P. falciparum* genome reveals the presence of 15 and 10 orthologs related to the biosynthesis of GPI-anchors and *N*-glycans, respectively [35]. Many of these sequences had been identified in previous works, and most of them are annotated in the genome of the parasite (Table 1) [26], [32], [36]. The annotated genes encode for enzymes involved in each sequential step of the *N*-glycosylation and GPI biosynthetic processes (Fig. 1), including all the main catalytic subunits. Only ALG enzymes that add mannose or glucose to *N*-glycans [28], [31] and one regulatory subunit in each one of the three major multimeric complexes, namely the phosphatidylinositol *N*-acetylglucosaminyltransferase, the GPI-anchor transamidase and the OST, are missing in the parasite genome (labelled as NI in Table 1). Apart from those, all enzymes or enzymatic complexes required for the biosynthesis and transfer of *N*-glycans or GPI molecules to proteins are identifiable in the genome of *P. falciparum*. Notably, *P. falciparum* GPI anchors contain glycan core species with three or four mannose residues [10], [37], although genes encoding SMP3/PIGZ proteins required for the transference of a fourth mannose do not seem to be present in the genome. However, recent data indicate that GPI10/PIGB mannosyltransferase 3 may be involved in the addition of the terminal fourth mannose to the GPI glycan core [38]. Remarkably, all the genes identified show evidence of expression during asexual intraerythrocytic development and other life stages of the parasite life cycle [35]. Furthermore, most of the proteins (i.e. 14 out of 25) have been detected by mass spectrometry in proteomic experiments [35].

**Table 1:** *P. falciparum* annotated genes related to GPI-anchor biosynthesis and *N*-glycosylation.

Pathway	Enzyme/EC	Gene ID	Enzyme/subunit name	MIS^a^	MFS^b^	MS evidence*^c^*	Expression evidence (Asexuals)*^c^*	Expression evidence (Other stages)*^c^*
GPI anchor biosynthesis	Phosphatidylinositol *N*-acetylglucosaminyltransferase (EC 2.4.1.198)							
	Subunits	**PF3D7_1032400*^d^***	**PIGA/GPI3*^d^***	0.798	−2.212	N	Y	Y
		PF3D7_0618900	PIGQ/GPI1	0.254	−2.777	Y	Y	Y
		PF3D7_0911000	PIGC/GPI2	0.534	−2.452	N	Y	Y
		PF3D7_0935300	PIGP/GPI19	0.134	0	N	Y	Y
		PF3D7_1141400	PIGH/GPI15	0.226	−2.53	Y	Y	Y
		NI	PIGY/Eri1					
	*N*-acetylglucosaminyl-phosphatidylinositol de-N-acetylase (EC 3.5.1.89)	PF3D7_0624700	PIGL/GPI12	0.99	−2.574	Y	Y	Y
	GPI-anchored wall transfer protein 1 (EC:2.3.-.-)	PF3D7_0615300	PIGW/GWT1	0.133	−2.898	N	Y	Y
	GPI mannosyltransferase 1 (EC:2.4.1.-)	PF3D7_1210900	PIGM/GPI14	0.181	−2.588	N	Y	Y
	GPI mannosyltransferase 2 (EC:2.4.1.-)	PF3D7_1247300	PIGV/GPI18	0.121	−3.045	N	Y	Y
	GPI mannosyltransferase 3 (EC:2.4.1.-)	PF3D7_1341600	PIGB/GPI10	0.488	−2.397	N	Y	Y
	GPI ethanolamine phosphate transferase 3 (EC:2.7.-.-)	PF3D7_1214100	PIGO/GPI13	0.135	−2.876	Y	Y	Y
	GPI-anchor transamidase (EC:3.-.-.-)							
	Subunits	**PF3D7_1128700**	**PIGK/GPI8**	0.214	−2.706	Y	Y	Y
		PF3D7_1122100	PIGT/GPI16	0.137	−3.131	Y	Y	Y
		PF3D7_1369000	GPAA1 (or GAA1)	0.119	−3.007	Y	Y	Y
		PF3D7_1330700	PIGU/Gab1	0.143	−3.113	Y	Y	Y
		NI	PIGS/GPI17					
*N*-glycosylation	UDP-*N*-acetylglucosamine--dolichyl-phosphate N-acetylglucosamine phosphotransferase (EC:2.7.8.15)	PF3D7_0321200	ALG7	0.131	−2.94	N	Y	Y
	*N*-acetylglucosaminyldiphosphodolichol N-acetylglucosaminyltransferase (EC:2.4.1.141)							
	Subunits	**PF3D7_0806400**	**ALG13**	0.191	−3.406	Y	Y	Y
		PF3D7_0211600	ALG14	0.12	−3.101	Y	Y	Y
	Oligosaccharyltransferase (OST) (EC 2.4.1.119)							
	Subunits	**PF3D7_1116600**	**STT3**	0.917	−2.76	Y	Y	Y
		PF3D7_0919600	WBP1	0.137	−3.079	Y	Y	Y
		PF3D7_0311600	OST1	0.136	−3.075	Y	Y	Y
		PF3D7_0726800	OST2	0.143	−3.108	N	Y	Y
		PF3D7_1243200	OST5	0.794	−2.429	N	Y	Y
		PF3D7_0107700	OST3/OST6	0.479	−1.084	Y	Y	Y
		PF3D7_1233050	OST4	N/A	N/A	N	Y	Y
		NI	Swp1					

a Mutagenesis index score, based on the number of random transposon insertions in the gene in saturation mutagenesis studies (see [39]).

b Mutagenesis fitness score, a proxy of mutant growth fitness calculated by saturation mutagenesis analysis (see [39]).

c Mass Spec and Expression evidence obtained from PlasmoDB genomic database (see [35]).

d Predicted catalytic subunits in oligomeric complexes are shown in bold.

NI, Not Identified by similarity search; N/A, Not Available.

Previous data obtained by transposon-based large-scale genetic screening assays indicate that a majority of these genes play a significant biological role in the development of asexual intraerythrocytic stages [39]. Thus, most of the genes involved in the biosynthesis of GPI anchors and *N*-glycosylation, present low mutagenesis index scores (MIS), which display the probability of gene disruption in *piggyBac* transposon-based saturation mutagenesis, and do not show insertions within their coding sequences (CDS) [39]. Surprisingly, a single disruption was detected in both STT3, the catalytic subunit of OST, and PIGL/GPI12, *N*-acetylglucosaminyl-phosphatidylinositol de-*N*-acetylase (GlcNAc-PI de-*N*-acetylase). Nevertheless, all the single enzymes and catalytic subunits depicted in the table, including also STT3 and PIGL/GPI12, show a high fitness cost for *in vitro* growth, pointing out the importance of GPI-anchor biosynthesis and *N*-glycosylation for asexual intraerythrocytic parasite development [39].

### 2.2 GPI-biosynthesis inhibitors disrupt the growth of *P. falciparum* asexual parasites

**Fig. 2:**
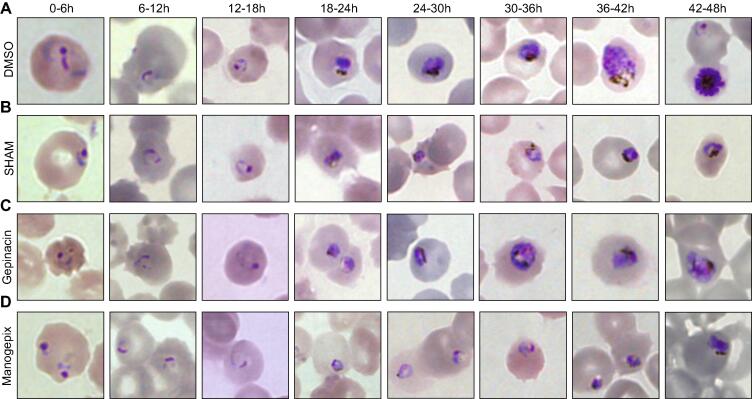
GPI inhibitors suppress *P. falciparum* 3D7 growth at trophozoite stages. Microscopy Giemsa-stained smears of tightly synchronized (5 h window) *P. falciparum* parasites growth in presence of: (A) DMSO (as a carrier control); (B) SHAM; (C) gepinacin; and (D), manogepix. Images show the effect of compounds on parasite development at different time intervals.

**Fig. 3:**
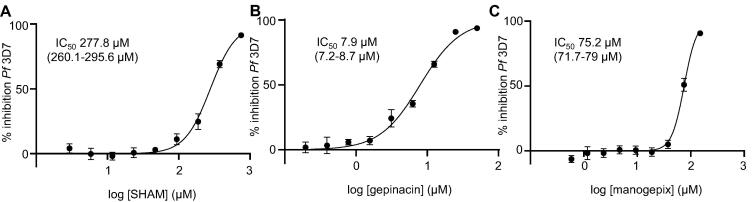
Dose-response curves of GPI inhibitors. Percentage of *P. falciparum* 3D7 inhibition caused by treatment with different concentrations of: (A) SHAM; (B) gepinacin; or (C) manogepix. Graphs and calculated IC_50_s are representative of three biological replicates. IC_50_ values calculated for each compound are indicated within each plot (with 95% confidence interval in brackets).

The relevance of GPI-anchors and *N*-glycans in many eukaryotic organisms, together with the presumed essentiality of many of the genes involved in their biosynthetic pathways in *P. falciparum* (Table 1), prompted us to investigate the effect of specific inhibitors upon the growth of asexual intraerythrocytic parasites in culture. Despite the paucity of molecules and the poor development of GPI targets, mostly due to the absence of robust 3D structures of the component proteins, recent studies describe the identification of selective compounds targeting the GPI pathway of pathogenic fungi [33], [34], [40]. Considering the structure of *P. falciparum* GPI anchors [10], together with the commercial availability of compounds, we analyzed the effect of manogepix [40] and gepinacin [34], both inhibitors of inositol acyltransferase GWT1, on the growth of *P. falciparum*. Likewise, we also tested salicylic hydroxamic acid (SHAM) as previous works had shown its activity as inhibitor of the *Trypanosoma brucei* GlcNAc-PI de-*N*-acetylase [41]. All three compounds distinctly halted the growth of *P. falciparum* during the trophozoite stage of intraerythrocytic asexual development (Fig. 2), showing varied -but rather modest- IC_50_s (Fig. 3). The paucity of inhibitors affecting the *N*-glycosylation drove us to focus on tunicamycin, a well-known inhibitor of ALG7, the first committed enzyme of the process [42]. As described before, tunicamycin did not affect the growth of the parasite during the first intraerythrocytic developmental cycle (IDC) (Supplementary Fig. S1) [43], [44]. Similar analyses were also carried out with *P. falciparum* DD2 multi-resistant parasites [45] and with HepG2 cells, to assess cytotoxicity (Supplementary Table S1).

### 2.3 *In silico* docking study of compounds targeting the GPI-biosynthetic pathway

A docking analysis was performed to confirm the inhibition of SHAM in PIGL, and manogepix and gepinacin in GWT1. Since no protein structures are available for these enzymes, models from the AlphaFold Protein Structure Database were used. Docking predictions using these models have already been tested [46], and they can be useful to explore protein–ligand binding modes. The quality of the models used along this work is summarized in Supplementary Figs. S2 and S3, indicating a significant degree of confidence in highly conserved regions which have been used as prospective binding pockets. All the residues inside the binding boxes showed a predicted local distance difference test (pLDDT) score above 70, indicating good backbone prediction, and in most cases these were even above 90, which hinted at a correct orientation of side chains (Supplementary Fig. S3) [46].

**Fig. 4:**
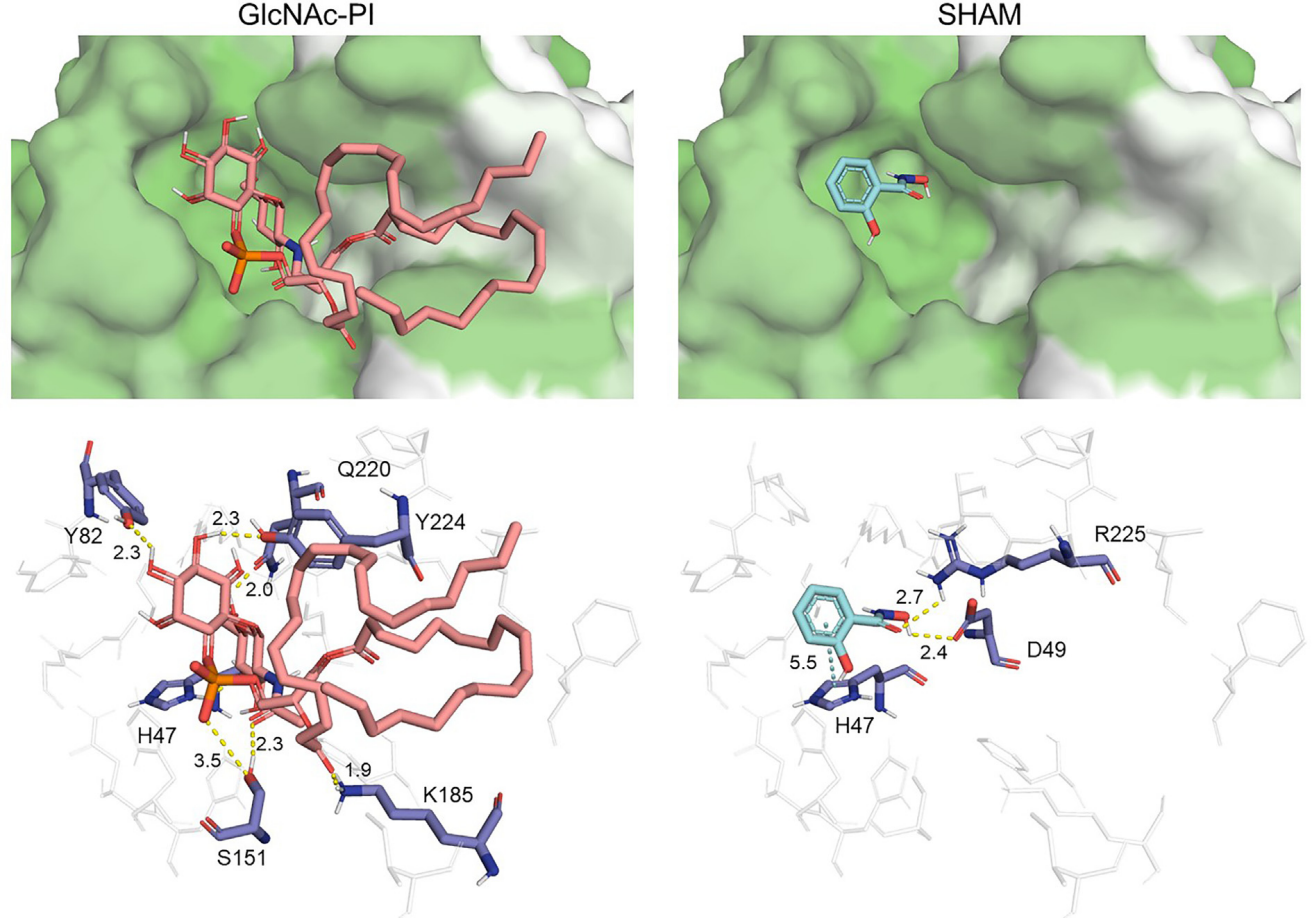
Binding of natural ligands and inhibitors on PIGL. Top panels show the hydrophobic surface of the proteins (white: more hydrophobic, green: less hydrophobic), and bottom panels illustrate the interactions between residues (dark blue) and molecules (pink: GlcNAc-PI; light blue: SHAM). (For interpretation of the references to colour in this figure legend, the reader is referred to the web version of this article.)

Predicted binding energies for PIGL docking were similar for GlcNAc-PI, the enzyme's natural ligand, and SHAM, with the latter having a slightly less negative value (Table 2). While the positioning of the SHAM molecule was similar to that described for *T. brucei* enzyme [41] (Fig. 4), the long and flexible GlcNAc-PI aliphatic tails could have impacted the binding of the ligand, as AutoDock Vina simulations with many torsions are discouraged. In this regard, the SHAM molecule was directly interacting with the supposedly catalytic D49, while the most common interaction produced by the simulations of the GlcNAc-PI acetyl group was with S151. Additionally, despite the high quality of the PIGL model, it naturally lacked the metal cofactor and the activated water of the catalytic site [47], which could have affected the predicted binding modes and energy of the molecules.

**Table 2:** Docking results for PIGL and GWT1. The mean (in Kcal/mol) and standard deviation for the predicted binding energy is calculated from the selected 100 best binding modes.

**Enzyme**	**Type**	**Ligand**	**ΔG mean(Kcal/mol)**	**ΔG deviation**
PIGL	Ligand	GlcNAc-PI	−6.10	0.30
PIGL	Inhibitor	SHAM	−6.00	0.01
GWT1	Ligand	GlcN-PI	−7.86	0.42
GWT1	Ligand	myristoyl-CoA	−8.05	0.54
GWT1	Inhibitor	gepinacin	−7.70	0.29
GWT1	Inhibitor	manogepix	−9.23	0.48

**Fig. 5:**
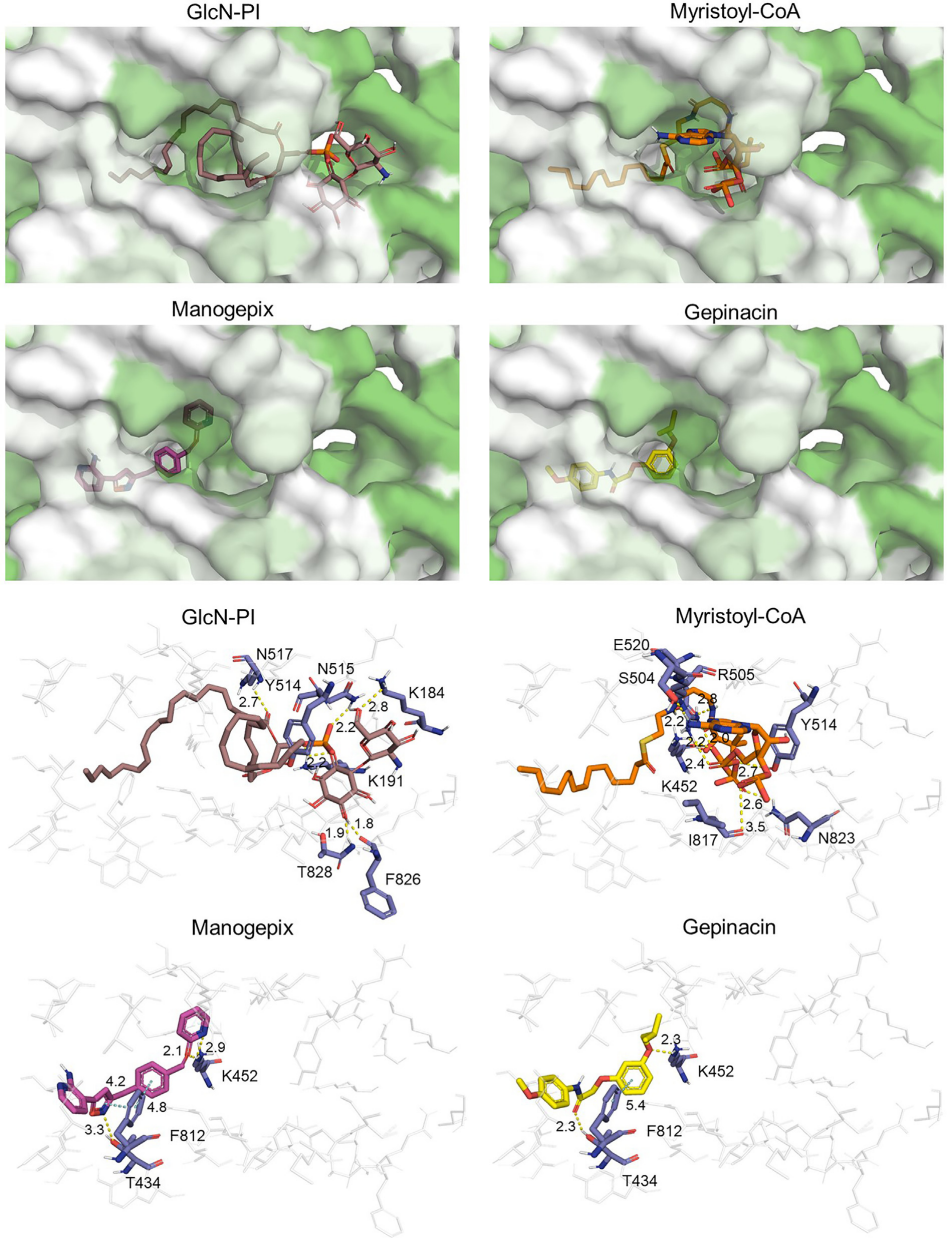
Binding of natural ligands and inhibitors on GWT1. Top panels show the hydrophobic surface of the proteins (white: more hydrophobic, green: less hydrophobic), and bottom panels illustrate the interactions between residues (dark blue) and molecules (grey: GlcN-PI; orange: myristoyl-CoA; yellow: gepinacin; purple: manogepix). (For interpretation of the references to colour in this figure legend, the reader is referred to the web version of this article.)

On the other hand, the quality of the GWT1 model was low overall (Supplementary Fig. S2), with large unstructured regions showing a low pLDDT score [48], and thus unreliable [49]. However, the central part of the enzyme, which overlapped with the binding site predicted by blind docking and residue conservation, showed an overall good model confidence, with some pLDDT scores above 90 (Supplementary Fig. S3). Predicted binding energies for manogepix and gepinacin were mostly favourable, although the latter showed a moderately worse energy in comparison with the natural ligands GlcN-PI and myristoyl-CoA. Still, these ligands’ long aliphatic tails and the poor model confidence could have compromised their correct binding. Interestingly, both inhibitors located to a highly hydrophobic tunnel composed by phenylalanines, leucines and isoleucines. In particular, pi interactions such as those with F812, and polar interactions with T434 and K452 could help stabilize both compounds. The location of both inhibitors would probably prevent the correct positioning of a fatty chain such as the myristic acid of the myristoyl-CoA molecule (Fig. 5).

### 2.4 Tunicamycin delayed death-like effect is not rescued by isoprenoid precursors

**Fig. 6:**
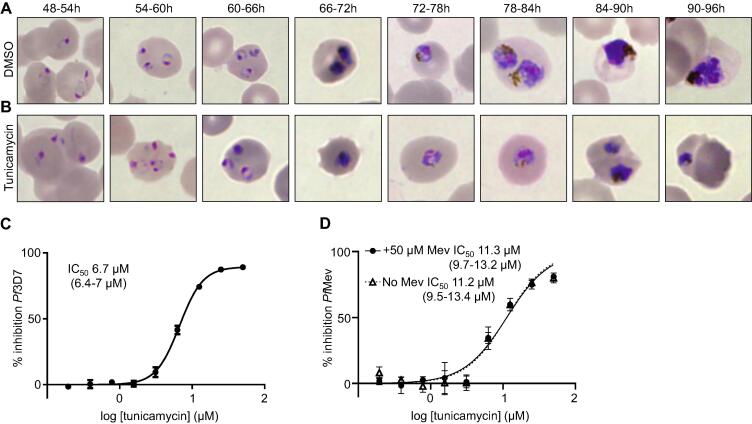
Tunicamycin halts *P. falciparum* 3D7 growth at the trophozoite stage during the second IDC after treatment. Microscopy Giemsa-stained smears of tightly synchronized (5 h window) *P. falciparum* parasites growth in presence of: (A), DMSO (as a carrier control); and (B) tunicamycin. Images show the effect of compounds on parasite development during the second IDC at different time intervals. (C) Dose-response curve of tunicamycin during the second IDC post-treatment on *P. falciparum* 3D7 parasites. (D) Dose response curve of tunicamycin during the second IDC post-treatment on *Pf*Mev parasites growth in presence (filled circles) or absence (open triangles) of 50 µM mevalonate. Graphs and calculated IC_50_s (including 95% confidence interval in brackets) are representative of three biological replicates.

Tunicamycin halts parasite development at the trophozoite stage during the second IDC after treatment (72–78 h, Fig. 6), as it had been reported before [43], [44]. This outcome strongly resembles the delayed death effect induced by inhibitors that selectively target the housekeeping functions of the apicoplast [50], [51], an essential plastid organelle whose key function is provide the parasite with isoprenoid precursors via the methylerythritol phosphate (MEP) pathway [52]. To assess whether tunicamycin induced delayed death effect was associated with isoprenoid synthesis, we used a *P. falciparum* strain engineered with the alternate mevalonate (MVA) pathway (*Pf*Mev) [53]. *Pf*Mev parasites are able to produce isoprenoid precursors when the culture media is supplemented with mevalonate, bypassing the lethal effect of compounds that inhibit the MEP isoprenoid pathway or disrupt the apicoplast [53]. However, the presence of mevalonate did not rescue *Pf*Mev parasites treated with tunicamycin. Therefore, this result strongly suggests that the tunicamycin induced delayed death phenotype is not related to the synthesis of isoprenoid precursors and the essential function of the apicoplast (Fig. 6).

### 2.5 Asexual blood stage inhibition profiling and structural modeling studies point to a specific effect of tunicamycin

**Fig. 7:**
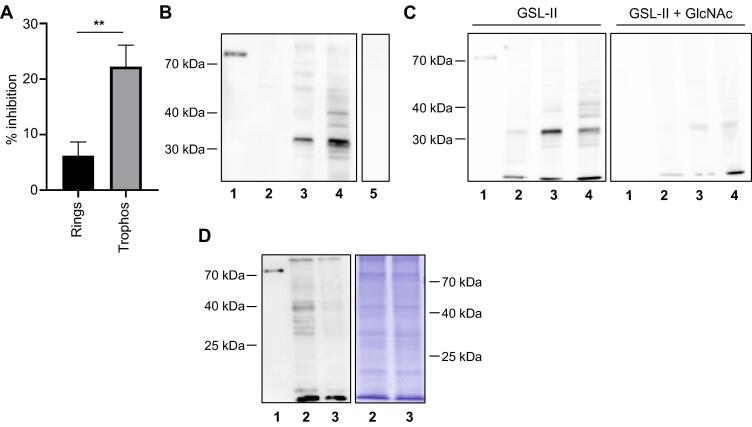
Tunicamycin delayed death is stronger in tight-synchronized trophozoites and reduces specific GSL-II labeling. (A) Selective treatment of 3 h-window rings (black bar) or trophozoites (grey bar) treated with tunicamycin show different inhibition percentages during the second IDC. Graph shows mean values ± SD from three replicates. Two-tailed unpaired Student *t*-test statistical significance (*p* < 0.01) is indicated by asterisks. (B) GlcNAc-binding lectin GSL-II blot with BSA-GlcNAc neoglycoprotein (1, Control) and extracts from *P. falciparum* rings (2), trophozoites (3), late trophozoites/schizonts (4) and uninfected red blood cells (5). (C) GSL-II lectin blot with BSA-GlcNAc (1, Control) and extracts from *P. falciparum* rings (2), trophozoites (3) and late trophozoites/schizonts (4), carried out with GSL-II pre-incubated without (left) or with 0.2 M of GlcNAc, to validate binding specificity. (D) GSL-II lectin blot with BSA-GlcNAc neoglycoprotein (1, Control) and extracts from *P. falciparum* trophozoites during the second IDC after DMSO (2) or tunicamycin treatment (3). A Coomassie-stained gel with DMSO (2) and tunicamycin treated (3) parasite extracts (right) is included as a loading control. (For interpretation of the references to colour in this figure legend, the reader is referred to the web version of this article.)

Contradictory data reported several years ago led to the notion that the effect of tunicamycin on parasite asexual growth was nonspecific and unrelated to the inhibition of *N*-glycan biosynthesis [43], [44], [54]. In order to verify this hypothesis, we inspected the inhibitory activity profile during the life cycle of *P. falciparum*. Our initial results indicated that the delayed death induced by tunicamycin was more significant when treatment was kept along mature stages, suggesting a stronger effect during this developmental phase (not shown). Tight parasite synchronization (3 h window) combined with selective 6 h treatments showed that growth inhibition was indeed significantly higher when parasites where exposed to tunicamycin during trophozoite development, emphasizing a more intense effect at this stage (Fig. 7), roughly matching ALG7 expression boost [55], [56]. Likewise, blots carried out with the GlcNAc-recognizing lectin GSL-II [11] showed a specific binding, which significantly increased in mature forms but was strikingly abrogated in the second IDC after tunicamycin treatment (Fig. 7).

**Fig. 8:**
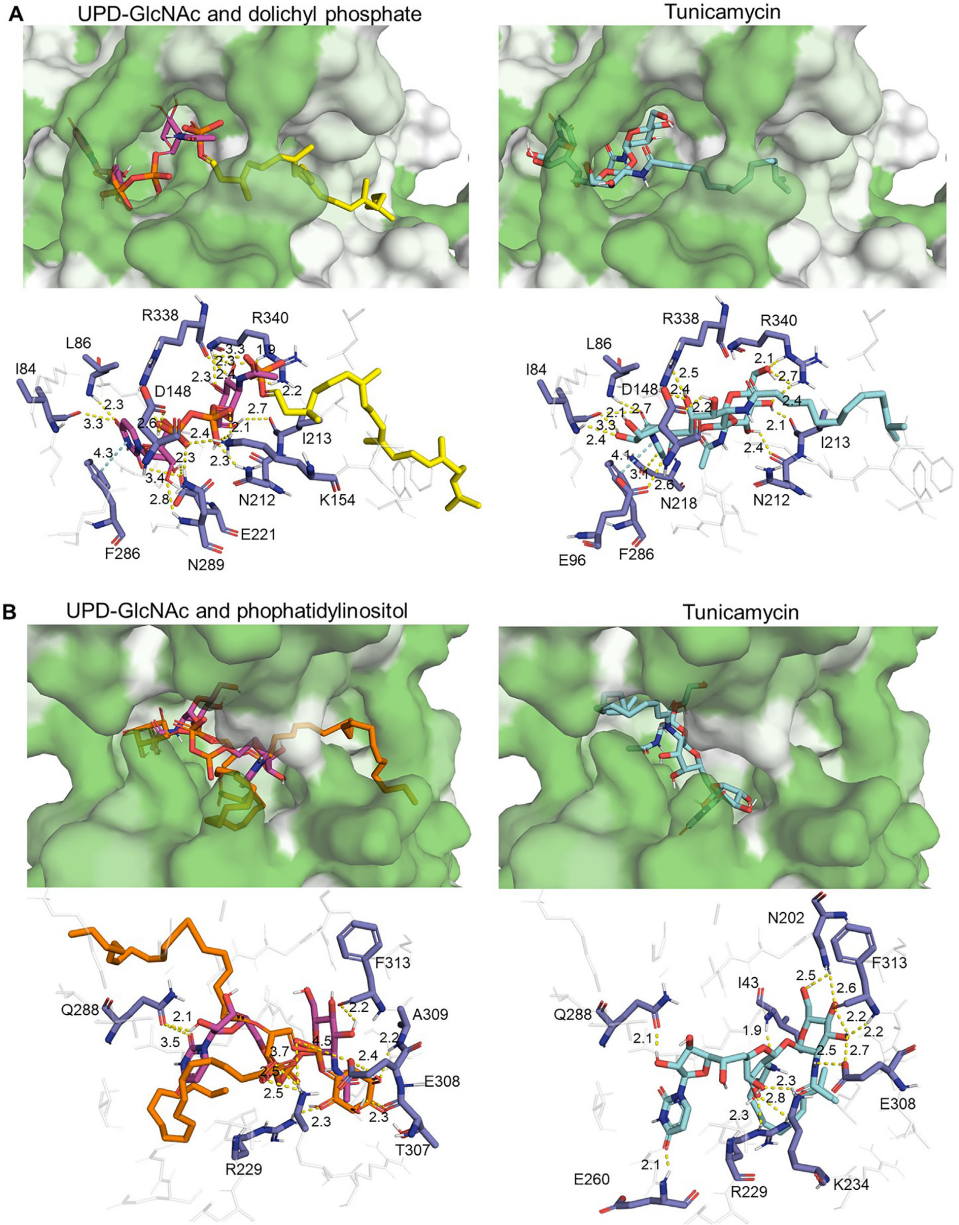
Binding of natural ligands and inhibitors on ALG7 (A) and PIGA (B). Top panels show the hydrophobic surface of the proteins (white: more hydrophobic, green: less hydrophobic), and bottom panels illustrate the interactions between residues (dark blue) and molecules (purple: UPD-GlcNAc; yellow: dolichyl phosphate; orange: phophatidylinositol; light blue: tunicamycin). (For interpretation of the references to colour in this figure legend, the reader is referred to the web version of this article.)

The AlphaFold-generated ALG7 model showed an overall high degree of confidence (Supplementary Figs. S2 and S3), and docking simulations suggest that tunicamycin is positioned in *P. falciparum* in a similar fashion as in the human ortholog GPT [42], with its main body roughly overlapping the UDP-GlcNAc-binding site and its aliphatic tail inserted into a hydrophobic grove (Fig. 8A). This binding position would prevent correct binding of both the UDP-GlcNAc and the dolichol phosphate [42]. Both tunicamycin and UDP-GlcNAc are stabilized by their uracil thanks to pi interactions with F286, and the highly hydrophilic R340 interacts with the GlcNAc moieties. Interestingly, the *N*-acetyl group does not appear to interact directly with any residue in tunicamycin, while in UDP-GlcNAc it appears to be near R338. Predicted binding energies showed a better binding energy for tunicamycin compared to the natural ligands, although the difference with UDP-GlcNAc was minor (Table 3). A docking simulation of tunicamycin against PIGA, the only other *N*-acetylglucosaminyltransferase identified in the genome of *P. falciparum*, suggests a lower binding affinity of the drug in comparison to that of UDP-GlcNAc. This would point to ALG7 as the most probable target of tunicamycin in the parasite.

**Table 3:** Docking results for ALG7 and PIGA. The mean (in Kcal/mol) and standard deviation for the predicted binding energy is calculated from the selected 100 best binding modes.

**Enzyme**	**Type**	**Ligand**	**ΔG mean(Kcal/mol)**	**ΔG deviation**
ALG7	Ligand	UDP-GlcNAc	−9,67	0,08
ALG7	Ligand	dolichyl phosphate	−7,19	0,37
ALG7	Inhibitor	tunicamycin	−9,91	0,18
PIGA	Ligand	UDP-GlcNAc	−9,27	0,25
PIGA	Ligand	phosphatidylinositol	−6,68	0,24
PIGA	Inhibitor	tunicamycin	−8,43	0,23

## 3 Discussion

Despite the rising number of publications casting new light on the glycobiology of the malaria parasite [12], [13], [14], [15], [16], [18], [57], [58], [59], there is an apparent scarcity of carbohydrate-active enzymes conserved in *P. falciparum* genome [9], especially when compared to other protozoan parasites [8]. Thus, apart from severely truncated *N*-glycans [11] and minor *O*- and *C*-glycans modifying key proteins [12], [15], the highly abundant GPI glycolipids seem to be the main glycans present in the surface of the parasite [10], at least in the asexual blood stages [20], [60]. Accordingly, the complex biosynthetic machineries of GPI-anchors and *N*-glycans can be distinctly identified in the genome [28], [32]. Both GPIs and *N*-glycans require UDP-*N*-acetylglucosamine (UDP-GlcNAc) as a precursor, which is generated through a classical amino sugar metabolic route [17] recently proved to be essential in intraerythrocytic asexual parasites [61], [62]. The importance of UDP-GlcNAc and the amino sugar pathway in *P. falciparum* [63], but also in murine models of malaria [64], is possibly associated to the high relevance of either or both glycan structures. The unequivocal effect of specific inhibitors affecting GPI-anchors or *N*-glycan biosynthesis on *P. falciparum* growth, albeit with high IC_50_ values, offers new insight into these metabolic pathways, which could be further exploited for the design of new antimalarials. Nevertheless, the high IC_50_ -also against multi-drug resistant parasites-, together with their comparable toxicity against HepG2, discourages further development of these compounds as antimalarial molecules.

As stated, GPIs are the most prominent glycoconjugates present in the asexual intraerythrocytic stages of *P. falciparum* [65], anchoring several key proteins to the surface of the parasite [66]. Furthermore, free GPI glycolipids -not bound to any protein- act as proinflammatory toxins contributing to the severity of malaria [67], [68]. Other essential proteins present in different stages of parasite development, such as Pfs48/45, Pfs25, Pfs28 and CSP, are also predicted to be GPI anchored [22], [69], [70]. In summary, these glycoconjugates play critical roles for the survival of the parasite along its complete life cycle, and previous works demonstrated the feasibility to devise specific GPI inhibitors in a *P. falciparum* cell free system [71]. Hence, GPI biosynthesis is deemed as an attractive target for the development of compounds against several pathogens, although the lack of 3D structural models of the enzymes in the biosynthetic pathway poses an important barrier to rational inhibitor design [72], [73], [74]. In recent years several works reported the identification of new compounds selectively targeting the GPI pathway in fungi and protozoan parasites [34], [40], [41]. The treatment of *P. falciparum* cultures with SHAM, manogepix or gepinacin rapidly halted the growth of parasites during the trophozoite stage of the parasite, roughly coinciding with the prominent 30 – 35 h boost of expression of their respective ortholog targets, GlcNAc-PI de-*N*-acetylase PIGL/GPI12 for SHAM, and inositol acyltransferase PIGW/GWT1 for manogepix and gepinacin [55], [56]. Remarkably, this timing also matched the increase of expression of most asexual GPI-anchored proteins [66], although it also has to be considered that new permeability pathways, which may improve compound uptake, are fully developed during trophozoite stages. Furthermore, despite the difficulties to obtain robust structural models of some of the enzymes, like PIGW/GWT1, docking analyses suggest the specific interaction of the inhibitors with the target *P. falciparum* proteins. Thus, the results confirm the relevance of GPI biosynthesis for *P. falciparum* and encourage the exploration of the pathway for the design of new specific and improved inhibitors against the parasite, also focusing in other stages of its development.

The presence of an active and prominent *N*-glycosylation mechanism in the asexual intraerythrocytic stages of *P. falciparum* remained initially a matter of scientific discussion, based mostly in the use of different metabolic labeling procedures [54], [65], [75], [76]. Enzymes involved in the synthesis of truncated *N*-glycan precursors, a feature also common in other protozoan parasites, do exist and are expressed in the parasite genome [28], [31], together with a complete OST complex that includes a STT3 catalytic subunit [32]. Furthermore, despite glycopeptides containing one or two GlcNAc *N*-linked glycans have never been unequivocally identified in malaria proteins, the detection of short lipid-linked glycan precursors and the labeling of specific membranes and organelles with the GlcNAc-binding lectin GSL-II seems to settle the issue [11]. Notably, the aforementioned *N*-glycosylation controversy also extended to the effect of tunicamycin on the growth of asexual parasite stages, reported several years ago [43], [44], [54]. In this regard, most of the *N*-glycosylation related genes, including the target of tunicamycin ALG7, show a high fitness cost which matches with the inhibitory effect of tunicamycin [39]. The docking analyses carried out, together with the stronger inhibition after tunicamycin treatment in mature stages and the abrogation of GSL-II labeling, strongly support the specific effect of this compound blocking *N*-glycosylation. The docking simulations using the high-quality AlphaFold model for ALG7 hint at the similarity of the mode of action of tunicamycin in *P. falciparum* and the human GPT [42]. Intriguingly, the delayed-death effect induced by this inhibitor, also described in the apicomplexan parasite *Toxoplasma gondii* [77], does not seem to be related to the generation of isoprenoid precursors, such as isopentenyl pyrophosphate (IPP) [51], [53]. IPP is the key metabolite supplied by the apicoplast and required for the survival of asexual parasites [52]. Hence, this result poses new interesting questions linked to the action of tunicamycin and the biological function of *N*-glycosylation, which may still be associated to the internal traffic of vesicles in the parasite [78]. Considering also the potential relevance of *N*-glycosylation in other parasitic stages, our results indicate that this pathway deserves further exploration since it may contribute to outline unexpected aspects of parasite biology.

## 4 Conclusion

All in all, by making use in *P. falciparum* cultures of known compounds recently characterized [34], [40], [41], [42], together with *in silico* docking studies based on newly available 3D molecular models [49], the data presented in this work contribute to set the stage for future exploitation of largely unexplored metabolic routes for antimalarial research. Thus, the results confirm that GPI biosynthesis and *N*-glycosylation are required for asexual parasite growth and, hence, one or several enzymatic steps in these metabolic routes are anticipated to be essential for parasite development. Nevertheless, further studies will be required to completely understand the biology underlying the observed growth inhibition in asexual but also in other stages, and to begin to harness the full potential of targeting GPI and/or *N*-glycosylation to control parasite growth.

## 5 Material and methods

### 5.1 Parasite culture and maintenance

*P. falciparum* parasites were cultured with human B^+^ erythrocytes (3% hematocrit) in RPMI medium supplemented with Albumax and incubated at 37 °C in an atmosphere of 92% N_2_, 3% O_2_ and 5% CO_2_ using standard methods [79]. Human erythrocytes were purchased from the Banc de Sang i Teixits (Catalonia, Spain), after approval from the Comitè Ètic Investigació Clínica Hospital Clínic de Barcelona (HCB/2020/0051). Parasite growth was monitored by counting the infected erythrocytes in Giemsa-stain blood smears by light microscopy.

### 5.2 *P. falciparum* growth inhibition assay

*P. falciparum* growth was primarily analyzed in *P. falciparum* 3D7 parasites under three different concentrations (100, 10, and 1 mM) of manogepix (Enamine Ltd., Kyiv, Ukraine), gepinacin (Enamine Ltd., Kyiv, Ukraine) and salicylic hydroxamic acid (SHAM, Merck, Darmstadt, Germany). Conditions were tested in triplicate for every concentration and data shown are representative of three different biological replicates. The three compounds and tunicamycin (Merck, Darmstadt, Germany) were also analyzed via standard growth inhibition assays to calculate half maximal inhibitory concentration (IC_50_), carried out as described in [80]. Drugs were added from different stock solutions in dimethyl sulfoxide (DMSO) so that the percentage of the latter was always below 0.4 % per well. Specific multi-drug resistant (DD2) [45] or engineered (*Pf*Mev [53], from a NF54 background) parasite lines were used when required. Parasitemia was first adjusted to 0.2–0.8% rings after sorbitol synchronization. Two hundred microliters of parasite culture (1% hematocrit) were plated in 96-well microplates and incubated for 48 h or 96 h at 37 °C with serial dilutions of the tested compounds, in triplicate. *P. falciparum* parasites engineered with an alternate mevalonate pathway [53] were growth in presence or absence of 50 µM mevalonate in the culture medium. Parasitemia was determined by fluorescence-assisted cell sorting (FACS) in a BD LSRFortessa™ cell analyzer (Becton Dickinson, Franklin Lakes, United States of America), equipped with a BD high throughput sampler (HTS) [81]. Infected red blood cells (RBCs) were stained with SYTO 11 (Molecular Probes, Life Technologies) to a final concentration of 0.5 μM for cytometry analysis. Non-infected and infected non-treated RBCs, exposed to an equivalent percentage of DMSO as carried solvent were included in triplicate in every plate. The single-cell population was selected on a forward-side scattergram, and the green fluorescence from this population was detected. Parasitemia was expressed in % as the number of parasitized cells per 100 erythrocytes. IC_50_ values were determined with GraphPad Prism 8 software, using a non-linear regression analysis model. At least three biological replicates (including three technical replicates each) were carried out with each different compound.

### 5.3 Tight synchronization and treatment at different asexual stages.

Parasites were tightly synchronized at 3 to 5 h windows by combining Percoll and sorbitol. Images of parasite development treated with different compounds (IC_90_) were obtained in an Olympus IX51 inverted microscope, after 5 h parasite synchronization and Giemsa staining of blood smears. To determine the timing of tunicamycin peak activity along the asexual IDC, parasites were shortly exposed (6 h intervals) to maximal concentrations of tunicamycin, at the early ring (0–6 h, after sorbitol synchronization) or early trophozoite phase (24–30 h, after sorbitol synchronization) during the first IDC, before tunicamycin removal and washing (adapted from [82]). Parasite growth was then monitored by FACS, confirming tunicamycin growth inhibition after the second IDC (greater than96 h).

### 5.4 HepG2 toxicity assays

HepG2 cells were detached, centrifuged, and resuspended in DMEM without phenol red. Cell viability was checked upon cell counting with trypan blue staining. Then, cells were diluted at a concentration of 3.2 × 10^5^ cells per mL before adding 100 µL per well to the 96-well plate. Each run contained its own negative (untreated cells) and positive (medium alone) controls [80]. Plates were incubated at 37 °C for 2 days. Assay readout was made by adding 50 µL per well of a PBS solution containing 10% alamarBlue reagent (Thermo Fisher Scientific); then, plates were incubated for another 6 h at 37 °C, before recording the fluorescence intensity with a Tecan Infinite M Nano + reader (excitation: 530 nm, emission: 590 nm) [80].

### 5.5 Parasite protein extracts

*P. falciparum* cultures were sorbitol synchronized and growth normally, or treated with IC_90_ tunicamycin concentrations or DMSO during the first IDC. Then, cultures were washed and collected after 30 h (trophozoite stage). Briefly, parasites were centrifuged for 5 min at 1500 rpm and the pellets resuspended in 2 RBCs volumes of 0.2% saponin in 1x PBS (phosphate buffered saline) to disrupt RBC membranes. Uninfected RBCs were used as controls. The suspensions were incubated for 10 min on ice, then 10 mL of 1x PBS were added and centrifuged at 4 °C for 8 min at 1800 rpm. Supernatant was removed and saponin lysis was repeated. After centrifugation, the pellet was washed with 1x PBS, transferred to a 1.5 mL tube and centrifuged at 4 °C for 10 min at 14,000 rpm. Pellets were kept at −80 °C before total protein extraction. Frozen pellets were thoroughly resuspended with lysis buffer, containing 1% sodium dodecyl sulfate (SDS), 20 mM Tris-HCl pH 7.4, 150 mM NaCl, 1 mM ethylenediaminetetraacetic acid (EDTA), and 1x EDTA-free Protease Inhibitor Cocktail (Merck, Darmstadt, Germany). Between 100 and 250 μL of lysis buffer was added, depending on the total number of parasites. Pellets were then resuspended and sonicated on ice three times for 10 s at 100% amplitude. After sonication, suspensions were centrifuged at 14 °C for 30 min at 14,000 rpm and supernatants containing the extracted soluble proteins were kept in 1.5 mL tubes and stored at −80 °C before protein quantification and lectin blot analysis.

### 5.6 *Griffonia simplicifolia* II (GSL-II) lectin blot

Parasite extracted proteins were analyzed by SDS-PAGE and GSL-II lectin blotting. Samples were loaded in 10% sodium dodecyl sulfate polyacrylamide electrophoresis gel (SDS-PAGE), together with a pre-stained protein ladder and 125 or 250 ng of a BSA GlcNAc-containing neoglycoprotein (Dextra Laboratories), as positive control. After gel electrophoresis, proteins were wet transferred to polyvinylidene difluoride (PVDF) membranes overnight at 4 °C. After the transference, the membrane was blocked using 3% BSA in TBST (50 mM Tris pH 7.4, 150 mM NaCl, 0.1% Tween 20) for 1 h. Then, the membrane was incubated with GSL-II lectin (1:400, Vector Labs) in 0.5% BSA in TBST (supplemented with 1 mM Ca^2+^ and 1 mM Mn^2+^) for 1 h, and washed 3 times for 5 to 10 min with TBST. For specificity assays, GSL-II was pre-incubated with GlcNAc 0.2 M for 30 min at RT. Finally, the membrane was incubated with NeutrAvidin-HRP (1:2000, ThermoFisher) in 0.5% BSA in TBST for 1 h before being rinsed 3 times for 5 to 10 min with TBST and a final wash with TBS (50 mM Tris pH 7.4, 150 mM NaCl) for 5 to 10 min. All incubations were performed under gentle constant agitation at room temperature. For assay readout Pierce ECL Western Blotting Substrate (ThermoFisher, Waltham, USA) was added on the membrane following the manufacturer instructions. A LAS4000 imaging system was used to analyze chemiluminescence derived from detected protein bands.

### 5.7 Computational methods

PlasmoDB [35] was consulted in order to obtain information about the genes involved in the biosynthesis of *N*-glycans and GPI-anchors in *P. falciparum* 3D7, including any transcriptomic and proteomic evidence and their phenotypical characteristics by mutagenesis experiments (Table 1). Protein models for ALG7 (PF3D7_0321200), PIGA (PF3D7_1032400), PIGL (PF3D7_0624700) and GWT1 (PF3D7_0615300) of *P. falciparum* were downloaded as PDB files from the AlphaFold Protein Structure Database on July 23, 2021 [49]. Charges and polar hydrogens were added using AutoDockTools 1.5.6. [83], and the resulting structures were formatted as PDBQT files. Structures for natural ligands and inhibitors were obtained as SDF files from PubChem [84] as follows: tunicamycin (CID 16,220,051 2D), gepinacin (CID 2,337,633 3D), manogepix (CID 16,719,049 3D), SHAM (CID 66,644 3D), UDP-GlcNAc (CID 445,675 3D), dolichyl phosphate (CID 24,892,715 2D), phosphatidylinositol (CID 71,581,204 2D), GlcNAc (CID 439,174 3D), GlcN (CID 439,213 3D) and myristoyl-CoA (CID 11,966,124 2D). Structures with only 2D data were first processed in Avogadro 1.2.0 [85] in order to optimize their molecular geometry and obtain the lowest-energy 3D conformers. Structures for GlcNAc-PI and GlcN-PI were also generated in Avogadro from phophatidylinositol and GlcNAc or GlcN, respectively. All 3D structures were later processed with AutoDockTools to generate PDBQT ligand files.

Clustal Omega [86] was used to compute multiple sequence alignments for PIGA, PIGL and GWT1 (Supplementary Fig. S4). Ortholog sequences from *H. sapiens*, *M. musculus*, *S. cerevisiae*, *C. albicans*, *T. gondii* and *P. vivax* were identified via OrthoMCL-DB [87] and downloaded from UniProt [88]. Docking was done with AutoDock Vina 1.1.2 [89]. For ALG7, the binding box was determined using the human GPT in complex with tunicamycin as model (Protein Data Bank entry 6BW5) [42], [90]. For PIGA, PIGL and GWT1, the binding box was determined by previous blind docking, residue conservation and/or literature [47] (Supplementary Fig. S3). Energy range was set to 4 and exhaustiveness to 8. A total of 100 docking rounds with different random seeds were performed for each enzyme and their natural ligands and inhibitors, each round producing nine different binding modes from which the one with the lowest binding energy (in Kcal/mol) was kept. The means and standard deviations of the binding energies were obtained from the selected 100 best binding modes. PyMOL 2.4.1 [91] was used to visualize and render images of the docking results. Hydrophobicity surface maps were generated using the Kyte–Doolittle hydrophobicity scale [92].

## Funding

We acknowledge funding from the Spanish Ministry of Science & Innovation, R + D + i Grant PID2019-110810-I00 and the European Union Horizon 2020 Marie Sklodowska-Curie Action GA 703305. We would also like to thank the support of Carlos III Health Institute RICET Network for Cooperative Research in Tropical Diseases through the project RD12/0018/0010 (co-funded by European Regional Development Fund/European Social Fund “Investing in your future”).

## CRediT authorship contribution statement

**Àngel Fenollar:** Investigation, Validation, Formal analysis, Methodology, Writing – review & editing. **Albert Ros-Lucas:** Software, Formal analysis, Writing – review & editing, Visualization. **María Pía Alberione:** Investigation, Writing – review & editing. **Nieves Martínez-Peinado:** Investigation, Methodology, Writing – review & editing, Visualization. **Miriam Ramírez:** Investigation, Validation, Writing – review & editing. **Miguel Ángel Rosales-Motos:** Investigation, Validation. **Ling Yen Lee:** Investigation, Validation. **Julio Alonso-Padilla:** Writing – review & editing. **Luis Izquierdo:** Conceptualization, Methodology, Writing – original draft.

## Declaration of Competing Interest

The authors declare that they have no known competing financial interests or personal relationships that could have appeared to influence the work reported in this paper.
