# Supplementary figures and images for: Compounds targeting GPI biosynthesis or N-glycosylation are active against Plasmodium falciparum
Source: Comput Struct Biotechnol J. 2022 Feb 2;20:850–63. doi: 10.1016/j.csbj.2022.01.029 (PMC8841962; doi:10.1016/j.csbj.2022.01.029)

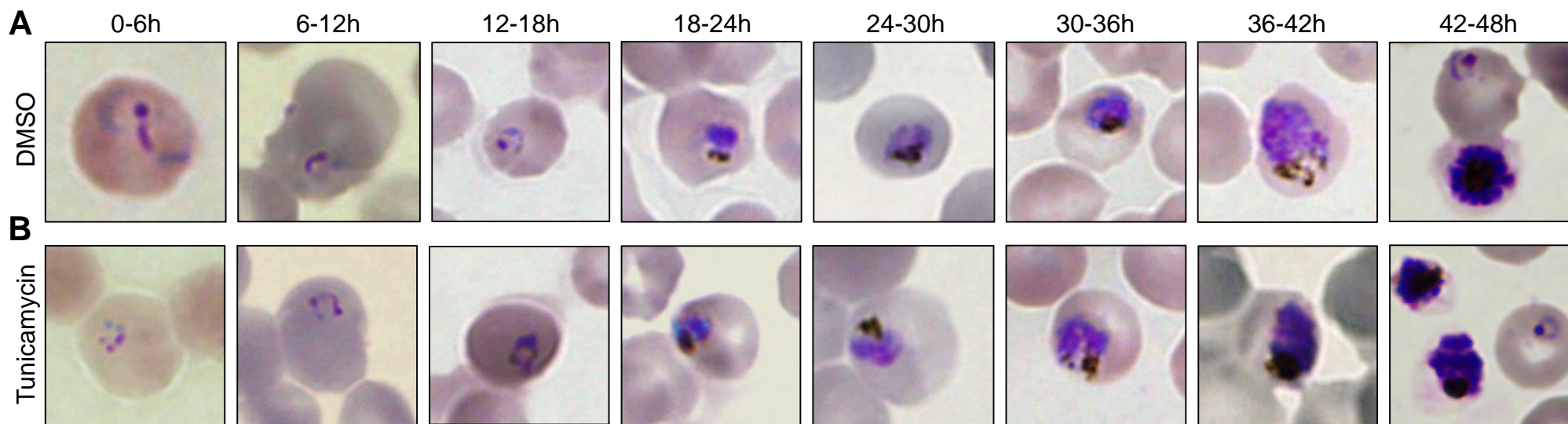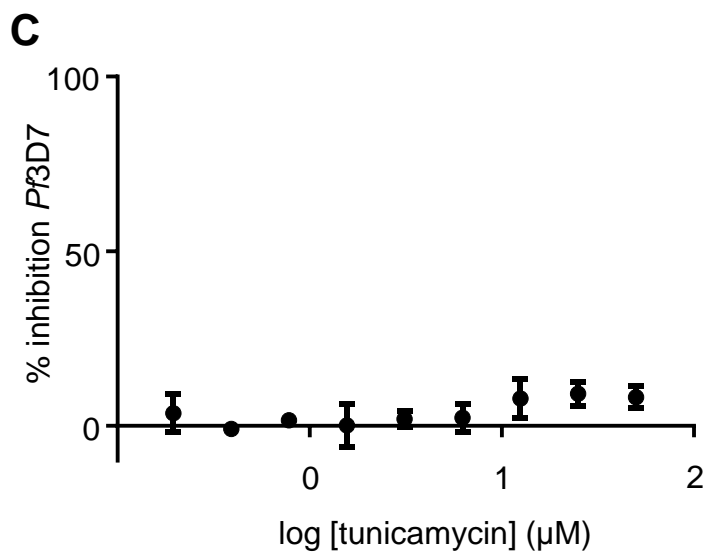

Supplement: Supplementary data 1 [file mmc1.pdf]

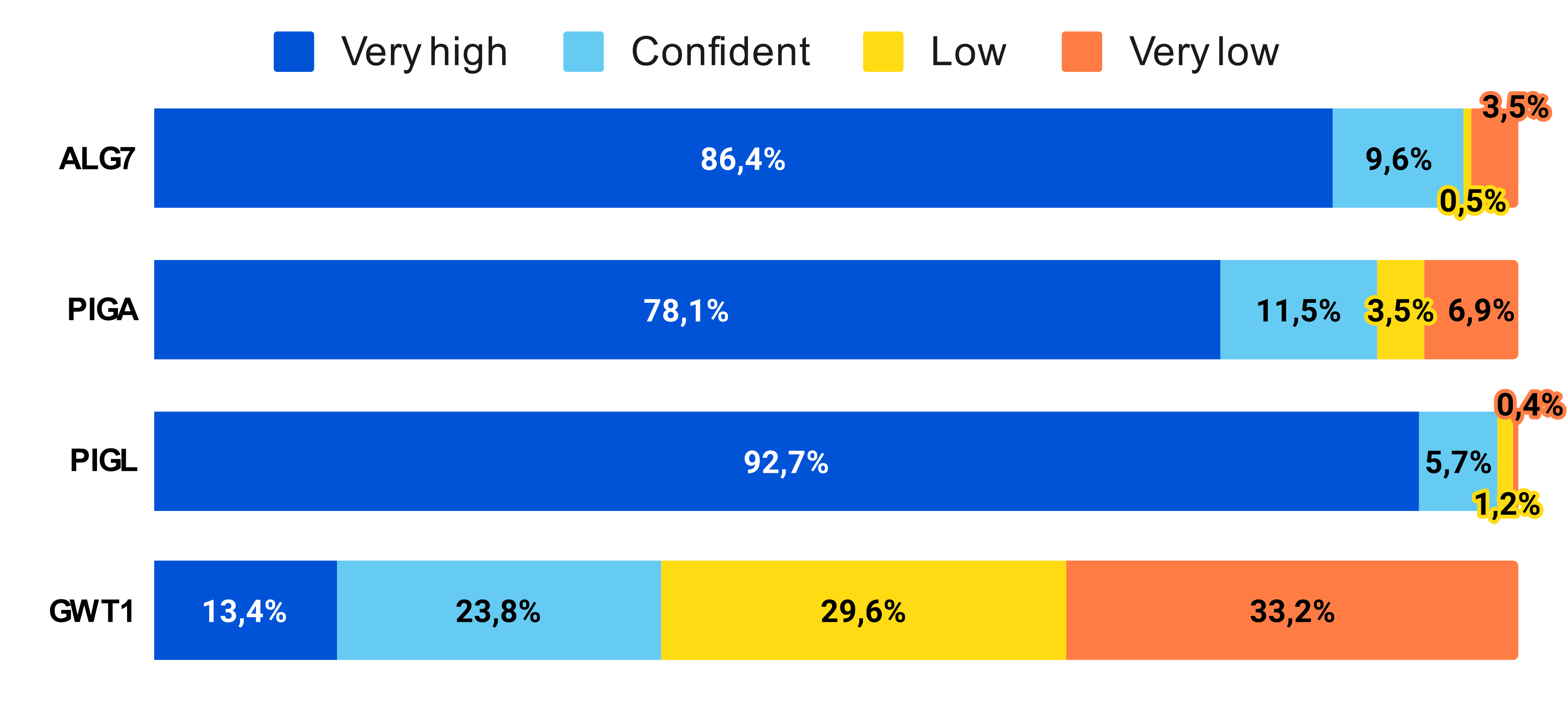

Supplement: Supplementary data 2 [file mmc2.zip › Supplementary Figure S2.png]

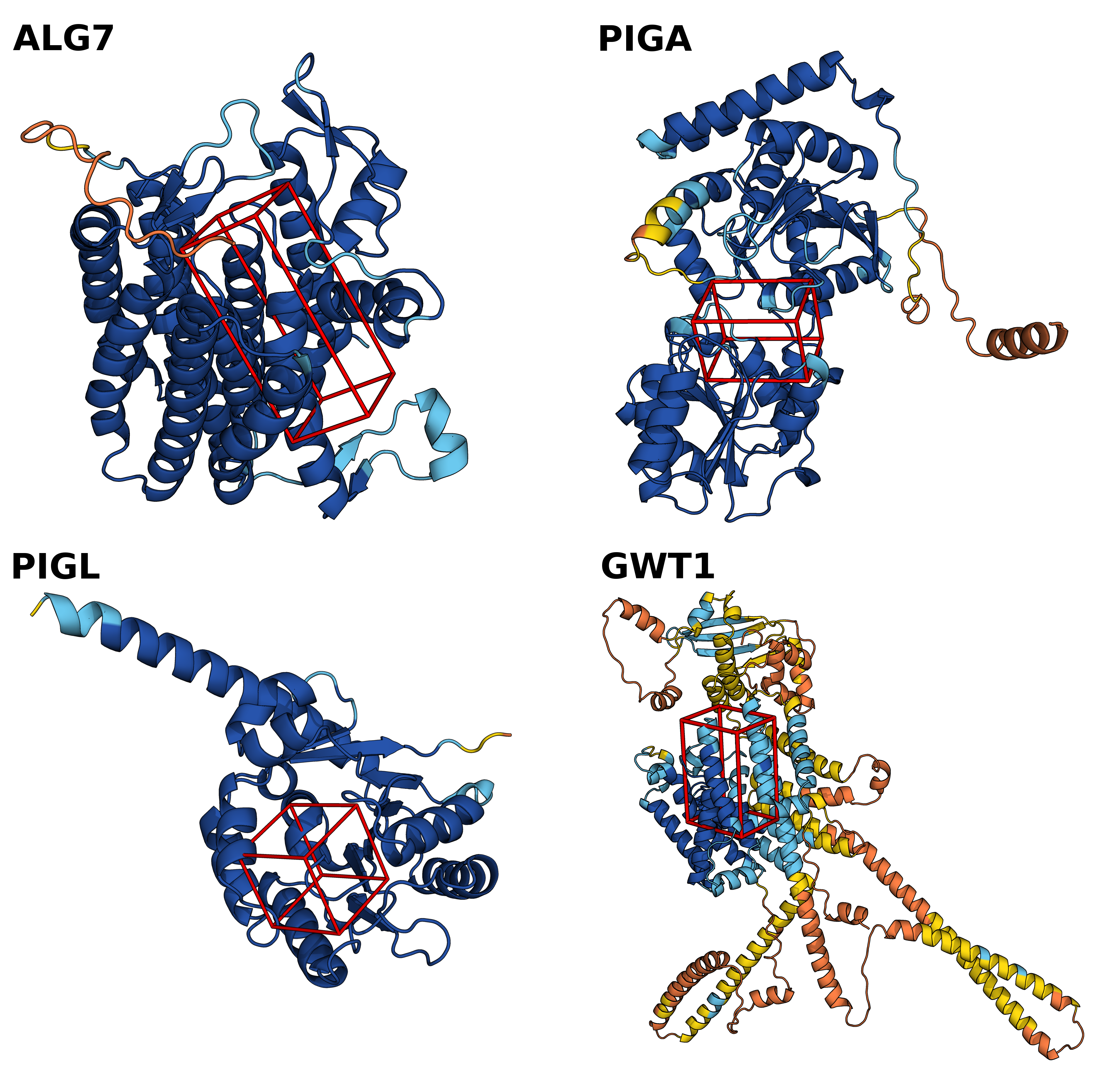

Supplement: Supplementary data 3 [file mmc3.zip › Supplementary Figure S3.png]
