# Supplementary material for: Compounds targeting GPI biosynthesis or N-glycosylation are active against Plasmodium falciparum
Source: Comput Struct Biotechnol J. 2022 Feb 2;20:850–63. doi: 10.1016/j.csbj.2022.01.029 (PMC8841962; doi:10.1016/j.csbj.2022.01.029)
Supplement: Supplementary data 4 [file mmc4.pdf]

A. PIGA

```
H. sapiens (P37287)          ------MACRGGAGNGHRASATLSRVSPGSLYTCRTRTHNICMVSDFFYPNMGGVESHIY      54
M. musculus (Q64323)         ------MANRRGGGQGQPPSVSPSPGSSGNLSDDRTCTHNICMVSDFFYPNMGGVESHIY      54
S. cerevisae (P32363)        ------------------------------------MGFNIAMLCDFFYPQLGGVEFHIY      24
C. albicans (Q5A6R7)         ------------------------------------MGYNIAMVTDFFYPQPGGVEFHVY      24
T. gondii (Q867V4)           ------MEAPRGGG-SA-----------QASTRRRRQICICMVSDFFFPSLGGIETHIY      42
P. vivax (A0A1G4GUJ0)        MMGPLKTDPPTGTAERM-----------RSLVYRKERKCCICMVSDFFYPNLGGIETHIF      49
P. falciparum (Q8IJ83)       -MESAVSECNIYKKEKD-----------KNIIYKQERKCCICMVSDFFYPNLGGIETHIF      48
                                                                     *.*: ***:*. **:* *::

H. sapiens (P37287)          QLSQCLIERGHKVIIVTHAYGNRKGIRYLTSGLKVYYLPLKVMYNQSTATTLFHSLPLLR      114
M. musculus (Q64323)         QLSQCLIERGHKVITVTHAYGNRKGVRYLTNGLKVYYLPLRVMYNQSTATTLFHSLPLLR      114
S. cerevisae (P32363)        HLSQKLIDLGHSVVIITHAYKDRVGVRHLTNGLKVYHVPFFVIFRETTFPTVFSTFPIIR      84
C. albicans (Q5A6R7)         HLSQKLIELGHSVVIITHNYSSRNGVRVLTNGLKVYYVPLWVIYRSSVFPTVFSCFPILR      84
T. gondii (Q867V4)           HLSQCLIQRGYKVVAITHYTDGRHGVRYLSNGLKVYYLPFVPVHDNATLPTFFSFFPLIR      102
P. vivax (A0A1G4GUJ0)        ELSKQLIKKGFKVIVVTHCYNNRHGVRWMGNGIKVYYLPFETYMDVVTFPNIVGTLPLCR      109
P. falciparum (Q8IJ83)       ELSKNLIKKGFKVIVVTNFNNNRHGIRWMGNGIKVYYLPFQPFLDVVSFPNIIGTLPLCR      108
                             .**: **. *..*: :*:   .* *:* : .*:***::*:          ...  :*: *

H. sapiens (P37287)          YIFVRERVTIIHSHSSFSAMAHDALFHAKTMGLQTVFTDHSLFGFADVSSVLTNKLLTVS      174
M. musculus (Q64323)         YIFVRERITIIHSHSSFSAMAHDALFHAKTMGLQTVFTDHSLFGFADVSSVLTNKLLTVS      174
S. cerevisae (P32363)        NILLREQIQIVHSHGSASTFAHEGILHANTMGLRTVFTDHSLYGFNNLTSIWVNKLLTFT      144
C. albicans (Q5A6R7)         NIFIRENIEIIHGHGSFSTLCHEAILHGRTMGLKTVFTDHSLFGFAEIGSIMGNKALKFT      144
T. gondii (Q867V4)           NILLRERADIVHGHQATSPLAHEASLVARALGMHVVYTDHSLFGFADMACIHLNKVLRFV      162
P. vivax (A0A1G4GUJ0)        NILYRERVDIVHGHQATSALAHQFILHAKSLGLKTIYTDHSLYSFSDKGCIHVNKLLKYC      169
P. falciparum (Q8IJ83)       NILYREKVDIVHGHQATSALAHQFILHAKTLGIKTIYTDHSLYSFSDKGCIHVNKLLKYC      168
                              *: **.  *:*.* : * :.*:  : ..::*::.::*****:.* :  .:  ** *

H. sapiens (P37287)          LCDTNHIICVSYTSKENTVLRAALNPEIVSVIPNAVDPTDFTPDPFR------RHDSI-T      227
M. musculus (Q64323)         LCDTNHIICVSYTSKENTVLRAALNPEIVSVIPNAVDPTDFTPDPFR------RHDSVIT      228
S. cerevisae (P32363)        LTNIDRVICVSNTCKENMIVRTELSPDIISVIPNAVVSEDFKPRDPTGGTKRKQSRDKIV      204
C. albicans (Q5A6R7)         FSDVGHVICVSHTCKENTVLRGSIDPIKVSVIPNAVISKDFKPKSHCV---NKNYTKEIT      201
T. gondii (Q867V4)           LHDLDACICVSHTHRENFVLRAGVPPSRVYVINNAVDASTLVPDPSKR-----PKPPEIR      217
P. vivax (A0A1G4GUJ0)        INDVDHSICVSHTNRENLVLRTEINPYKTSVIGNALDTRKFVPCLSKR-----PKLPRIN      224
P. falciparum (Q8IJ83)       INDVDHSICVSHTNRENLVLRTESNPYKTSVIGNALDTTKFVPCISKR-----PKFPRIN      223
                             : : .  **** * :** ::*    *    ** **:    : *

H. sapiens (P37287)          IVVVSRLVYRKGIDLLSGIIPELCQKYPDLNFIIGGEGPKRIILEEVRERYQLHDRVRLL      287
M. musculus (Q64323)         VVVVSRLVYRKGTDLLSGIIPELCQKYQELHFLIGGEGPKRIILEEVRERYQLHDRVQLL      288
S. cerevisae (P32363)        IVVIGRLFPNKGSDLLTRIIPKVCSSHEDVEFIVAGDGPKFIDFQQMIESHRLQKRVQLL      264
C. albicans (Q5A6R7)         IVVITRLFPNKGADLLTAVIPKICQLKPKVKFLIAGDGPKFLDLEQMREKYFLQERVTLV      261
T. gondii (Q867V4)           VVVLSRLTYRKGIDLLVTVIPPICKKLPNVNFVIGGYGPKRIILEEMREKHGLQDRVELI      277
P. vivax (A0A1G4GUJ0)        VIVISRLTYRKGVDLIAKVIPLVCHKYPFIKFIIGGDGPKRVLLEEMRERNHLHNSVVLL      284
P. falciparum (Q8IJ83)       IIVISRLTYRKGIDLIVKVIPLVCQKYPFIKFIIGGEGPKRLLLEEMREKYHLHNSVVLL      283
                             ::*: **  .** **:  :** :*     :.*::.* *** : :::: *   *:. * *:

H. sapiens (P37287)          GALEHKDVRNVLVQGHIFLNTSLTEAFCMAIVEAASCGLQVVSTRVGGIPEVLPENLIIL      347
M. musculus (Q64323)         GALEHKDVRNVLVQGHIFLNTSLTEAFCMAIVEAASCGLQVVSTKVGGIPEVLPESLIIL      348
S. cerevisae (P32363)        GSVPHEKVRDVLCQGDIYLHASLTEAFGTILVEAASCNLLIVTTQVGGIPEVLPNEMTVY      324
C. albicans (Q5A6R7)         GAIKHEEVRDVMVQGDIYLHPSLTEAFGTVIVEAASCGLYVVTTKVGGIPEVLPNEMTSF      321
T. gondii (Q867V4)           GAVSHDKVCALLQSGHIFLNTSLTESFCIAIVEAAACGMLVVSTNVGGIPEVLPPHMVLL      337
P. vivax (A0A1G4GUJ0)        GKVKQENVKNVLQTGHIFLNASLTEAFCIAIIEAASCGLLVISTDVGGISEVLPHDMMIL      344
P. falciparum (Q8IJ83)       GKVKQENVKNILQTGHIFLNTSLTEAFCIAIIEAASCGLLVISTDVGGISEVLPHDMMIL      343
                             * : :..*  ::  *.*:*: ****:*   ::***:*.: :::* **** ****  :

H. sapiens (P37287)          CEPSVK-SLCEGLEKAIFQLKSGTLPAPENIHNIVKTFYTWRNVAERTEKVYDRVSVEAV      406
M. musculus (Q64323)         CEPSVK-SLCDGLEKAIFQVKSGTLPAPENIHNVVKTFYTWRNVAERTEKVYERVSKETV      407
S. cerevisae (P32363)        AEQTSVSDLVQATNKAINIIRSKALD-TSSFHDSVSKMYDWMDVAKRTVEIYTNISSTSS      383
C. albicans (Q5A6R7)         AEPEEN-SLIDAAIDAINKIESNEID-TSKFHDAVAKMYSWNDIARRTENVYNSLDLDKL      379
T. gondii (Q867V4)           SEPDDV-QVTRRLEEAISIVHTV--D-PFSFHEQIREHYSWHDVAARTERVYFSLLFPEH      393
P. vivax (A0A1G4GUJ0)        AKPNHL-DLCAAVDSALERLKHV--D-SQAFHERLTKMYSWEKVAEKTEKVYMDVLSYAN      400
P. falciparum (Q8IJ83)       AKPNHI-ELCKAVDKALKIVQKV--D-SNLFHERLTKMYSWEKVAEKTEKVYMNVLNYAN      399
                             .:     .:     .*:  :.         :*: :   * * .:* :* .:*  :

H. sapiens (P37287)          L-----------------------------------------------------------      407
M. musculus (Q64323)         L-----------------------------------------------------------      408
S. cerevisae (P32363)        A-----------------------------------------------------------      384
C. albicans (Q5A6R7)         N-----------------------------------------------------------      380
T. gondii (Q867V4)           ASYPPCSASSPLAAPCNCGHAPLCLEEDEGQLQESAPAEDCVASGEQHWQREGRHPDAGQ      453
P. vivax (A0A1G4GUJ0)        P-----------------------------------------------------------      401
P. falciparum (Q8IJ83)       P-----------------------------------------------------------      400
```

```
H. sapiens (P37287)         -----------------------------------------------PMD---KRLDRL      416
M. musculus (Q64323)        -----------------------------------------------PMH---KRLDRL      417
S. cerevisae (P32363)       -----------------------------------------------DDKDWMKMVANL      396
C. albicans (Q5A6R7)        -----------------------------------------------ES---LLHRLQR      389
T. gondii (Q867V4)          ACREAGLVPRSLSGKTTNSFYEDSLCQCCRQPPLCCGCLLLPPSPPPLPSPFIVVQRLRK      513
P. vivax (A0A1G4GUJ0)       ----------------------------------------------------TILSRIKK      409
P. falciparum (Q8IJ83)      ----------------------------------------------------SLFNRIRK      408

H. sapiens (P37287)         ISHCGPVTGYIFALLAVFNFLFLIFLRWMTPDSIIDVAIDATGPRGAWTNNYSHSKRGGE      476
M. musculus (Q64323)        ISHCGPVTGYMFALLAVLSYLFLIFLQWMTPDSFIDVAIDATGPRRAWTHQWPRDKKRDE      477
S. cerevisae (P32363)       YKRDGIWAKHLYLLCGIVEYMLFFLLEWLYPRDEIDLAP-----------KWPKKTVSNE      445
C. albicans (Q5A6R7)        YYCCGIIAGKLYALCVIVDIFIFVILEWLYPADHIDKAT-----------KWPSAIKEED      438
T. gondii (Q867V4)          IYELGPVSGKIFCIVAILTWVYIRILEFFSPSAEIEEAP-----------AFPCDLLLHL      562
P. vivax (A0A1G4GUJ0)       IYDINTVFSKVYIFIIMLSYISCRLLEWLKPREDIEEAI-----------SFPHFIEED-      457
P. falciparum (Q8IJ83)      IYEINTVFSIIYIFIIMISYIGCQILEWLMPRQNIEEVV-----------SFPHFYDDEN      457
                            .     :: :  :.  .   :*.:: *   *: .            :

H. sapiens (P37287)         NNEISE-TR------------------------------------------         484
M. musculus (Q64323)        NDKISQ-SR------------------------------------------         485
S. cerevisae (P32363)       TKEARE-T-------------------------------------------         452
C. albicans (Q5A6R7)        ESEEET-FIFP--------NKVN----------------------------         452
T. gondii (Q867V4)          SEDSADQDGLQARKRTEREERVEVKESAGECICSKSTAHTPWAAEANAASTGGE         616
P. vivax (A0A1G4GUJ0)       ---------------------------------------------------         457
P. falciparum (Q8IJ83)      KNEK-----------------------------------------------         461
```

B.PIGL

```
H. sapiens (Q9Y2B2)         -----------------------------------MEAM-----WLLCVALAV------L      14
M. musculus (Q5SX19)        -----------------------------------MELV-----GFLCVAVAV------L      14
S. cerevisae (P23797)       -----------------------MKMLRR-TKVNFSKLL-----YKITKLAIV------L      25
C. albicans (C4YD13)        -----------------------------------MIFKLPLILLRLYITSF-------I      18
T. gondii (S8F741)          MERIQRRLAQTSSPSHSRFLWGPLQLLSVHSDLDVFLLLLPVLSLAVCLLSILLHAQASH      60
P. vivax (A0A564ZX95)       ----------------------------------MQHLSCPC---ACAWTGVVLL--LGG      21
P. falciparum (C6KT83)      ----------------------------------MSYYIPL---AI----IFTI--FSL      16

H. sapiens (Q9Y2B2)         AWGFLWVWDSSERMK---------S----------------------------REQGG--      35
M. musculus (Q5SX19)        TWGFLRVWNSAERMR---------S----------------------------PEQAG--      35
S. cerevisae (P23797)       --TILYIYFTP-KIV---------S----------------------------RNNASLQ      45
C. albicans (C4YD13)        IWIFQTTILPQTLTKLTNVSIKTQTFQHYYPYT---------------SLVNTPKN----      59
T. gondii (S8F741)          ARRFLLELLNRKGSDVGAVASSCVERNEEKPDEEAKKRGKETHSAQKQGDEAHGENKEES      120
P. vivax (A0A564ZX95)       VFYFLWHYLSERKK----------------------------------------------      35
P. falciparum (C6KT83)      ILYITINYLNKKKY----------------------------------------------      30
                               :

H. sapiens (Q9Y2B2)         ----RLGAESRTLLVIAHPDDEAMFFAPTVLGLARLR---HWVYLLCFSAGNYY-NQGET      87
M. musculus (Q5SX19)        ----LPGAGSRALVVIAHPDDEAMFFAPTMLGLARLE---QQVSLLCFSSGNYY-NQGEI      87
S. cerevisae (P23797)       HIFPHKYGDYEINLVIAHPDDEVMFFSPIISQLNSYFPRTVPFNIICLSKGNAE-GLGET      104
C. albicans (C4YD13)        SNSINIITNSNITYIIAHPDDEVMFFAPSIIELKK-PKYNNQINLICFSKGNYIKSMDEI      118
T. gondii (S8F741)          PTEKKRNNRLKVALVVAHPDDEVMFFTPTLALLRD-FSEHVQVHLLCLSTGNAA-GLGRV      178
P. vivax (A0A564ZX95)       DLLTWLKEKENVSFVIAHPDDEIMFFFPTIKLLFE-KKKKEEIFLLSLTNGDFY-SQGKI      93
P. falciparum (C6KT83)      N-FSNLLGNKNISIIVAHPDDELMFFFPTIKFLFD-KKKKNIFLLCLSNGNYY-GYGNI      87
                                      .   ::****** *** * :  *          . ::.:: *:   . ..

H. sapiens (Q9Y2B2)         RKKELLQSCDVLGIPLSSVMIIDNRDFPDDPGMQWDTEHVARVLLQHIE-------VNGI      140
M. musculus (Q5SX19)        RKKELLQSCAVLGIPPSRVMIIDKRDFPDDPEVQWDTELVASTLLQHIH-------ANGT      140
S. cerevisae (P23797)       RVRELNESAALLL-HNERAVSVQVMDFQDGMDEIWDIDSITSSLSQKID-------IKNH      156
C. albicans (C4YD13)        RQSELIQSSRILGIDQV-----SILDYQDGMNETWQLNDIVQSLHENLSPTSSGSDTNSN      173
T. gondii (S8F741)          RSREFLNAARLFGVENGNALVLDDEALQDGWG-LWSPERVADVVEEFIE-------KNEI      230
P. vivax (A0A564ZX95)       REKELYHVWSYLGGVKNNCKVMNDPNVQDGSA-LWNDQHLADILADYCT-------RCNI      145
P. falciparum (C6KT83)      REQELYKVWSYIGGEKNNCHIWNDNKIQDGWL-YWDEKYIFKLIKDYCI-------QYDI      139
                            *  *: .    :           .     *.    *. . :   : :

H. sapiens (Q9Y2B2)         ---NLVVTFDAGGVSGHSNHIALYAAVRALHSEGKLPKGCSV------------------      179
M. musculus (Q5SX19)        ---DLVVTFDAEGVSGHSNHIALYKAVRALHSGGKLPKGCSV------------------      179
S. cerevisae (P23797)       NLNQIIVTFDSYGVSNHINHKSCYAAVKKLVDDYAQPKTKRN------------------      198
C. albicans (C4YD13)        NKPSVLITFDDQGVSNHPNHISLFHGTKKYIQELRRKSENKNKTRIKT------------      221
T. gondii (S8F741)          ---STIFTFDERGVSRHPNHISVFRGVRLLHERQRARGNARPGGSERQRAEGKDERGGVT      287
P. vivax (A0A564ZX95)       ---SNVLTFDGYGVSGHPNHISVHRSARLLSKR---------------------------      175
P. falciparum (C6KT83)      ---KTIFTFDNYGVSGHPNHISAYKSIRMLSHM---------------------------      169
                               . :.***  *** * ** : . . :
```

```
H. sapiens (Q9Y2B2)            -------------------LTLQS--VNVLRKYISL----LDLPLSLLHTQ---------    205
M. musculus (Q5SX19)           -------------------LTLQS--VNALRKYAFL----LDLPWTLLSPQ---------    205
S. cerevisae (P23797)          -----------EQPPHVTALYLRSYKNNIVLKYNSFIWEILKILYDLISPFRRIIQALP-    246
C. albicans (C4YD13)           -TTTTINYIDIDSSSSTKFYVLKS--LNFFEKYSFTILGNIEILFNYISLLIKKFININI    278
T. gondii (S8F741)             KRGRHSRERGETRMSQTDVYLLQS--HGLFRKYMGV----LDVILSTVACRKK-------    334
P. vivax (A0A564ZX95)          --------------MGIKVLTLHS--THLIVKYLGM----FSLPFLL-------------    202
P. falciparum (C6KT83)         --------------KDIDIYTLKS--TNIIYKYMSF----FSYPFIT-------------    196
                                                    *:*     . **       :.

H. sapiens (Q9Y2B2)            --------------------------DVLFVLN--SKEVAQAKKAMSCHRSQLLWFRRLY    237
M. musculus (Q5SX19)           --------------------------DVLFVLT--SKEVAQAKKAMSCHRSQLLWFRYLY    237
S. cerevisae (P23797)          -----PN-----TAA---------EKDKLSLMNTHAQYVLAFATMLNAHESQVVWFRYGW    287
C. albicans (C4YD13)           NVSFFSNQIIKSKFDNNNNNLQLQNLNDIRFYSDLNMLSLSYAAMAYGHFSQMVWFRYAW    338
T. gondii (S8F741)             -----PN-----RI------------------ASVKLTPFLSIRGMSAHWSQFVWFRWMF    366
P. vivax (A0A564ZX95)          -----QK-----RF------------------LTFRFNPVLLLRLMYLYRSQFVYYRILF    234
P. falciparum (C6KT83)         -----NK-----RY------------------VIWSFNPLLLLRLMFFYKSQLVYYRILF    228
                                                                               : **.:::*  :


H. sapiens (Q9Y2B2)            IIFSRYMRINSLSFL----          252
M. musculus (Q5SX19)           VLFSRYMRINSLRFL----          252
S. cerevisae (P23797)          WIFSRFVFVNEFDVYTY--          304
C. albicans (C4YD13)           LLLSRYLTYNHLIEQ----          353
T. gondii (S8F741)             VFFSSYTYTNVFDRITV--          383
P. vivax (A0A564ZX95)          CLFSQYAYFNAFDLIS---          250
P. falciparum (C6KT83)         CIFSQYVYFNTFDLLKTYK          247
                                ::* :   * :
```

C. GWT1

```
H. sapiens (Q7Z7B1)            ------------------------------------------------------------    0
M. musculus (Q8C398)           ------------------------------------------------------------    0
S. cerevisae (P47026)          ------------------------------------------------------------    0
C. albicans (Q873N2)           ------------------------------------------------------------    0
T. gondii (S8ESW8)             MGGTPAAASPLQEARMRSPSCLSARDSFLASASPLRKVLFALFFSIGVALLLLLRLPFPQ    60
P. vivax (A0A564ZXH5)          ------------------------------------------------------------    0
P. falciparum (C6KSZ4)         ------------------------------------------------------------    0


H. sapiens (Q7Z7B1)            ------------------------------------------------------------    0
M. musculus (Q8C398)           ------------------------------------------------------------    0
S. cerevisae (P47026)          ------------------------------------------------------------    0
C. albicans (Q873N2)           ------------------------------------------------------------    0
T. gondii (S8ESW8)             TVVAPSPSSSLPTSLSLVHPLLPFFSLGSSDSLLALPASAPAETSSSSPSASPSAASVAP    120
P. vivax (A0A564ZXH5)          ------------------------------------------------------------    0
P. falciparum (C6KSZ4)         ------------------------------------------------------------    0


H. sapiens (Q7Z7B1)            ------------------------------------------------------------    0
M. musculus (Q8C398)           ------------------------------------------------------------    0
S. cerevisae (P47026)          ------------------------------------------------------------    0
C. albicans (Q873N2)           ------------------------------------------------------------    0
T. gondii (S8ESW8)             WWLPWSLSAGPAWWVSCPADTARRLGTPKAPRQLP---------GFGRRSRRTPQKAAEV    171
P. vivax (A0A564ZXH5)          ----MAHLNLLVYLIMCPFNVRHMLDAPSFPFRLGSKAASGETFTYGATARENLGS----    52
P. falciparum (C6KSZ4)         ----MSNMNILAYLLICPFNLIYIFDLPSYIPELNKKLENDEVFIYGKEIRKNESA----    52


H. sapiens (Q7Z7B1)            ------------------------------------------------------------    0
M. musculus (Q8C398)           ------------------------------------------------------------    0
S. cerevisae (P47026)          ------------------------------------------------------------    0
C. albicans (Q873N2)           ------------------------------------------------------------    0
T. gondii (S8ESW8)             PDAPSEAEEEAEAEEKEGRFRDGIAAAVPLQARWEHRLPESARQALAV-FALQGET----    226
P. vivax (A0A564ZXH5)          ---YSPAHDELYMLELAKMYYQ-------IVLTYKKDVRKGQEESYNLVVGSFGKEAK--    100
P. falciparum (C6KSZ4)         ---YSL-HYEKYLYELSRRYYE-------IILKYNKELGVNQEKEYNLIISREIDKKKKK    101


H. sapiens (Q7Z7B1)            ------------------------------------------------------------    0
M. musculus (Q8C398)           ------------------------------------------------------------    0
S. cerevisae (P47026)          ------------------------------------------------------------    0
C. albicans (Q873N2)           ------------------------------------------------------------    0
T. gondii (S8ESW8)             -ENNQAEEEERRRKRTWTCSNSGE----------RSELYDALQEVAFSNYEFPPFLEVFL    275
P. vivax (A0A564ZXH5)          ----------------------------GEVSLQRVLITNDAVYLSYQDVQNERGIQVKI    132
P. falciparum (C6KSZ4)         QKNSTQGEYNNDDDNNWKLFQIYEKEEPRSYELIRVEIYKKDILLIYKNEKTKSSIKFII    161
```

```
H. sapiens (Q7Z7B1)       ------------------------MSEKQMKEAFVSNLNGTTVLEITQGLCFP---AFCI    33
M. musculus (Q8C398)      ------------------------MSQKQLKEAFVRNLSGTSVLEVTQGLCFP---AFCI    33
S. cerevisae (P47026)     -----------------------MSTLKQRKEDFVTGLNGGSITEINAVTSIA---LVTY    34
C. albicans (Q873N2)      ----------------------MSSSLKQLKEQFVSDLTGGTIEEIYAVTSIA---LSSY    35
T. gondii (S8ESW8)        CVTLINLSVW-MFNAFSRCVASFSAWRSQRFSRFG-PPSGNSTSEASDLSETPSASAVAA    333
P. vivax (A0A564ZXH5)     K-R-GEISSYLDLLSWDSCLYKLNSDDYNLM----KSASDHSKPMVVSTYHIYMLLLVFS    186
P. falciparum (C6KSZ4)    K-KRKDIKNYF-SLCYQNCINKLDKNDYNIL----KSTINNSKENIINSAYIYMYIIFFF    215
                                                      :          . :

H. sapiens (Q7Z7B1)       LCRGFLIIF-------------------------SQYLCSFSP-TWKT----RFLTDFVV    63
M. musculus (Q8C398)      LCRGLWIIF-------------------------SQHVCSFSN-TWST----RFLMDFVV    63
S. cerevisae (P47026)     IS---WNLL-------------------------KNSNLMPPG-ISSV----QYIIDFAL    61
C. albicans (Q873N2)      LS---FRLL-------------------------KKS-------LGDL----ALIYDYIL    56
T. gondii (S8ESW8)        SSSSYARVRRRPPCASGATASFASPFASTSSQKSSVDASPPSP-RSHLSLLVNFLVNFAL    392
P. vivax (A0A564ZXH5)     LC------------TYVE----------------KSLLLEFPALKK-C----QVFLTLCL    213
P. falciparum (C6KSZ4)    LC------------IYVE----------------KNLFLYFPILLQKY----EILTTLFI    243
                           .                                .                   :    :

H. sapiens (Q7Z7B1)       LIVPMVATLTIWAS-------FILLEL-LGVIIFGAGLLYQ----IYRR----------R    101
M. musculus (Q8C398)      LIVPLVITLTVLSS-------FILLEN-LTVIVWGAWLLYQ----IYHR----------R    101
S. cerevisae (P47026)     NWVALLLSITIYASEPYLLNTLILLPC-LLAFIYGK-----------FT----------S    99
C. albicans (Q873N2)      NVLTILASITVYSNSPSYLHYFIVIPS-LVIYLVNY-----------HV-----------    93
T. gondii (S8ESW8)        LVLPLLLGFCLPNA---LLPVSVGVGSLTVAFLLPSWIR-R----IFQL-----------    433
P. vivax (A0A564ZXH5)     VYCPIISYLFFFYS----HV--SLL-GVLLVYVFFCGLFR--G---------------V    248
P. falciparum (C6KSZ4)    LFIPLILFVFFYFY----FTIIKLICSCLVLYVTFQLIYYTQGMPIYMEHSILKHKEKEE    299
                              ::  . .             :       :

H. sapiens (Q7Z7B1)       TCYARLP-----------------------------------------------------    108
M. musculus (Q8C398)      TCYAKVP-----------------------------------------------------    108
S. cerevisae (P47026)     SSKPSNP-----------------------------------------------------    106
C. albicans (Q873N2)      -EKPSSP-----------------------------------------------------    99
T. gondii (S8ESW8)        SETPKA------------------------------------------------------    439
P. vivax (A0A564ZXH5)     SCRRGGQ-----------------------------------HMGEQTGQH---------    264
P. falciparum (C6KSZ4)    ICDEKEEICDEKEEICDEKEEICDEKEEICDEKEEICDEKEEILDKKKKIHEKKKKIHDK    359


H. sapiens (Q7Z7B1)       ----------------------------------------FLKILEK---FLNISLESEY    125
M. musculus (Q8C398)      ----------------------------------------VQKVFAN---FLKISLESEY    125
S. cerevisae (P47026)     ----------------------------------------IYN-KKK---MITQRFQLEK    122
C. albicans (Q873N2)      ----------------------------------------HRQNDTK---EDKSDELLPR    116
T. gondii (S8ESW8)        -------------------------------------------VDA----SLFETDSCGR    452
P. vivax (A0A564ZXH5)     ----------------------------------TGDWHTIRGN------PQGDDTQEER    284
P. falciparum (C6KSZ4)    KEEIDEKKKKIHDKKDESHDKNEDITYPVQYNIENDLWYSSKNVDIKMYSSSNKGEEYII    419


H. sapiens (Q7Z7B1)       NPAISCFRVITSAFTAIAILAVDFPLFPRRFAKTELYGTGAMDFGVGGFVFGSAMVCLEV    185
M. musculus (Q8C398)      NPAITCYRVINSVFTAIAILAVDFPLFPRRFAKTELYGTGAMDFGVGGFIFGAAMVCPEV    185
S. cerevisae (P47026)     KPYITAYRGGMLILTAIAILAVDFPIFPRRFAKVETWGTSLMDLGVGSFVFSNGIVSSRA    182
C. albicans (Q873N2)      KQFITAYRSQMLIITNLAILAVDFPIFPRRFAKVETWGTSMMDLGVGSFVFSMGLANSRQ    176
T. gondii (S8ESW8)        ILALAEFRGALMIATCIAIYGVDFFIFPRSLAKTSAFGVSLMDLGVGCFVFSAGLVSRQA    512
P. vivax (A0A564ZXH5)     RKCLVHMRLANLCITYICIFAVDFYFFPRQFSKSFFFGNTLMDLGVGGCITSSAYSLNSK    344
P. falciparum (C6KSZ4)    QNTLKHFRLMNMCMTYICIFAVDFYFFPNHFCKSYYYGNTLMDIGIGASISSSAYSQEIK    479
                             :   *      * :.* .*** :**. :.*   :*   **:*:*  : . .

H. sapiens (Q7Z7B1)       RRRKYMEGSKL---------------------------------------HYFTNSLYSV    206
M. musculus (Q8C398)      RRK-SIEESRF---------------------------------------NYLRKSLYSV    205
S. cerevisae (P47026)     LLKNLSLKSKP------------------------------------SFLKNAFNALKSG    206
C. albicans (Q873N2)      LIKNHTDNYKFSW---------------------------------KSYLKTIKQNFIKS    203
T. gondii (S8ESW8)        RGEQRSKVAKGEQKHKETGGSATSLDGAADSRGKVEKVKSRKRNCLTRGVFTLLRAVGRS    572
P. vivax (A0A564ZXH5)     KLH--SANRKG------------------------------------------HLIDWKH    360
P. falciparum (C6KSZ4)    KFT--YIKEKK------------------------------------------RIIELKH    495
                                   :

H. sapiens (Q7Z7B1)       WPLVFLGIGRLAIIKSIGYQEHLTEYGVHWNFFFTIIVVKLITPLLLIIFPLNKSWIIAL    266
M. musculus (Q8C398)      WPLVFLGMGRLVIIKSIGYQEHSTEYGIHWNFFFTIIVVRLVTSLLLIIFPLNKSWIVAV    265
S. cerevisae (P47026)     GTLLFLGLLRLFFVKNLEYQEHVTEYGVHWNFFITLSLLPLVLTFIDPVTRMVPRCSIAI    266
C. albicans (Q873N2)      VPILVLGAIRFVSVKQLDYQEHETEYGIHWNFFFTLGFLPIVLGILDPVLNLVPRFIIGI    263
T. gondii (S8ESW8)        GVLGFAGILRFAAVSLLNYYTPVTEYGKHWNFYMSLMVLFIAAELLLPGSSSRPFLYVPM    632
P. vivax (A0A564ZXH5)     FILFFLGIARYIAVKLFNYNYSLTEYGMHWNFFLTLFFTLLTCNALLCLIRGVKR-TFHL    419
P. falciparum (C6KSZ4)    IVLFILGISRFIGIYLFNYNYNISEYGIHWNFFLTLCTTFLISNICFILLKRIRY-IFLF    554
                            : . *  *   :  : *    :*** ****::::    :                . .
```

```
H. sapiens (Q7Z7B1)          G--ITVLYQLALDFTSLKRLILYGTDGSGTRVGLLNANREGIISTLGYVAIHMAGVQTGL      324
M. musculus (Q8C398)         S--ITVVYQLALDYTPLKRILLYGTDGSGTRVGFLNANREGIISTLGYVTIHMAGVQTGL      323
S. cerevisae (P47026)        F--ISCIYEWLLLKDD--RTLNFLILA--DRNCFFSANREGIFSFLGYCSIFLWGQNTGF      320
C. albicans (Q873N2)         G--ISIAYEVALNKTG---LLKFILSSENRLESLITMNKEGIFSFIGYLCIFIIGQSFGS      318
T. gondii (S8ESW8)           GVALASVYQLLLWVAAAET---WV--LTADRDNFFTANREGILGCVGFFALYMIGVGVGS      687
P. vivax (A0A564ZXH5)        SCVLICLYEIIIWRLDIT---SYLVVDEAERSGFFSQNREGLMNVIGSVNLYLFSFSLWN      476
P. falciparum (C6KSZ4)       SIISIILFEIAIYYFDLH---NYILLK-NDRLNFFSSNKEGLFNIIGSVNLYLFSFSLFK      610
                                    ::  :          :          ::. *:**::. :*   :.: .

H. sapiens (Q7Z7B1)          YMHKNRSHIK--DLIKV-------------------------------------------      339
M. musculus (Q8C398)         YVLKGRAQVR--DWIKA-------------------------------------------      338
S. cerevisae (P47026)        YLLGNKPTLN--NLYKPST----------QD--------V--------------------      340
C. albicans (Q873N2)         FVLTGYKTKN--NLITISK----------IR--------I--------------------      338
T. gondii (S8ESW8)           LFFSAASLSS--RRPVSEE----------GE--------KGTAA----------------      711
P. vivax (A0A564ZXH5)        GYVFPD----------E------------GQQWERGKAERGKA-ARRP------------      501
P. falciparum (C6KSZ4)       YLTKQRTYITTSNIPKNKKDMNNSMYSKNGNHTNSNINNRNHKIVIRNNHINKYEQDNTN      670


H. sapiens (Q7Z7B1)          ------------------------------------------------------------      339
M. musculus (Q8C398)         ------------------------------------------------------------      338
S. cerevisae (P47026)        -----------------VA----------ASKKSSTWDYWTS-VTPLS--------G---      361
C. albicans (Q873N2)         -----------------SK----------KQHKKESSSFF-S-VATTQ--------GLYL      361
T. gondii (S8ESW8)           -----------------MK----------SQTVSRPPSRFAL-IAVLL--------GAA-      734
P. vivax (A0A564ZXH5)        --------------DEAART------PGEGHG---------------------QRSPARL      520
P. falciparum (C6KSZ4)       KYINKQINNNKNKLDEEEKLKKLKKLKNKKKNLKKKIKYYLLYLQYIINIYKEEYYTIYY      730


H. sapiens (Q7Z7B1)          ACFLLLAAISLFISLYV-VQVNVEAVSRRMANLAFCIWIVASSLILLSSLL-LGDIILSF      397
M. musculus (Q8C398)         TCWVFSVAVGFFISLHI-VQVNIEAVSRRMANLAFCLWVVASSLMLLSCLL-LSGIILSF      396
S. cerevisae (P47026)        LCIWSTIF--LVISQLV-FQYHPYSVSRRFANLPYTLWVITYNLLFLTGYC-LTDKIFGN      417
C. albicans (Q873N2)         ACIFYHLAFSLFISNLS----FLQPISRRLANFPYVMWVVSYNATFLLCYD-LIEKFIPG      416
T. gondii (S8ESW8)           VCF--------YLYLALVLAFYFDLLPTRRLINLPWVLLVAALNLYGLAGVL-LSEALVGR      785
P. vivax (A0A564ZXH5)        TLKLLLALSLLFHL-LHLLLNYYRRNYSVRILCNANYICVVSSVSLFAAALSYLVEKVLLRE      579
P. falciparum (C6KSZ4)       NIKLIISSFIFYL-LHIILNLYKNYSVRILCNANYIFLITSLGLFSCALSFSLEDILLRY      789
                                         :              * : *  :   : : .         :   :.

H. sapiens (Q7Z7B1)          AKFLIKGALVPCSWKLIQSPVTNKKHSESLVPEAERMEPSLCLITALNRKQLIFFLLSNI      457
M. musculus (Q8C398)         AQFLIKGSLVPCSWKLIQSPTTHKNHSESLILEAEKNQPSLCLITALNRNQLFFFLLSNI      456
S. cerevisae (P47026)        ---------------------------------SSEYYKVAECLESINSNGLFLFLLANV      444
C. albicans (Q873N2)         ---------------------------------N----LTSTVLDSINNNGLFIFLVSNL      439
T. gondii (S8ESW8)           G-----------------------------------PAGASYLVSGLSQNQIFIFLIANV      810
P. vivax (A0A564ZXH5)        K--------------------------------TT----TIPVLQQMNRHSLAVFLFCNV      603
P. falciparum (C6KSZ4)       K--------------------------------KYKINIDITVLDKINKNTLIVFLFSNI      817
                                                                        :  :. : : .**..*:

H. sapiens (Q7Z7B1)          TTGLINLMVDTLHSSTLWALFVVNLYMFSNCLIVYVLYLQDKTVQFW----------         504
M. musculus (Q8C398)         TTGLINLTMDTLHTGALWTLVVLSIYMFTNCLVIYVLDLQGKTIKFW----------         503
S. cerevisae (P47026)        STGLVNMSMVTIDSSPLKSFLVLLAYCSFIAVISVFLYRKRIFIKL-----------         490
C. albicans (Q873N2)         LTGFINMSINTLETSNKMAVIILIGYSLTWTLLALYLDKRKIYIKL-----------         485
T. gondii (S8ESW8)           LCGLTGLSMNRLLVPPALALSLLLLYALSWAFVAFGLGYLEKRIPLNL---------         858
P. vivax (A0A564ZXH5)        TMGTFNLLFQSLLFPLFFACLVLAAYSYGMLRFASLLPGPAQGEKGEKREKQ--Q--         656
P. falciparum (C6KSZ4)       LVGMFNILFQTLLLPLIFVIPILVFYSFLILLFTKCLPPSIRHPKKKTHHEEKQKKE         874
                               *  .: .  :         ::  *      .  *
```
