# Supplementary material for: Compounds targeting GPI biosynthesis or N-glycosylation are active against Plasmodium falciparum
Source: Comput Struct Biotechnol J. 2022 Feb 2;20:850–63. doi: 10.1016/j.csbj.2022.01.029 (PMC8841962; doi:10.1016/j.csbj.2022.01.029)
Supplement: Supplementary data 5 [file mmc5.docx]

Table S1. IC_50_ (µM) values in multidrug-resistant *P. falciparum* DD2 and TC_50_ (µM) values in HepG2 cells*^a^*.

| **Compound** | ***P. falciparum* 3D7*^b^*** | ***P. falciparum* DD2** | **HepG2 cells** |
| --- | --- | --- | --- |
| SHAM | 277.8 (260.1-295.6)*^c^* | 306.7 (273.5-346.2) | >750 (NA) |
| Gepinacin | 7.9 (7.2-8.7) | 35.3 (29.4-43.9) | >50 (NA) |
| Manogepix | 75.2 (71.7-79) | 67.6 (64-71.3) | 49.6 (40.7-60.7) |
| Tunicamycin | 6.7 (6.4-7) | 11.3 (9.9-13) | 5.7 (3.3-9.3) |

*a* Values are representative of three or more biological replicates

*b* *P. falciparum* 3D7 data, in the main text, is included in the table for comparison purposes

*c* 95% confidence interval is indicated in brackets
