# Supplementary material for: Compounds targeting GPI biosynthesis or N-glycosylation are active against Plasmodium falciparum
Source: Comput Struct Biotechnol J. 2022 Feb 2;20:850–63. doi: 10.1016/j.csbj.2022.01.029 (PMC8841962; doi:10.1016/j.csbj.2022.01.029)
Supplement: Supplementary data 6 [file mmc6.docx]

Supplementary Figure S1. Tunicamycin does not affect *P. falciparum* growth during the first IDC post-treatment. Microscopy Giemsa-stained smears of tightly synchronized (5 h window) *P. falciparum* parasites growth in presence of: (A), DMSO (as a carrier control); and (B) tunicamycin. Images show parasite development during the first IDC at different time intervals. (C) Dose-response curve of tunicamycin during the first IDC post-treatment.

Supplementary Figure S2. Per-residue model confidence of the studied enzymes, given by the predicted local distance difference test (pLDDT) score. Percentages represent the fraction of C_ɑ_ within each of the four categories. Very high: pLDDT ≥ 90, Confident: 90 > pLDDT ≥ 70, Low: 70 > pLDDT ≥ 50, Very low: 50 > pLDDT.

Supplementary Figure S3. Quality of the AlphaFold predicted-models given by the predicted local distance difference test (pLDDT) score of each residue. A: ALG7, B: PIGA, C: PIGL, D: GWT1. Dark blue: pLDDT ≥ 90 (very high), light blue: 90 > pLDDT ≥ 70 (confident), yellow: 70 > pLDDT ≥ 50 (low), red: 50 > pLDDT (very low). The red box represents the area used for ligand docking.

Supplementary Figure S4. Multiple sequence alignment of protein sequences of different species for the studied enzymes. A: PIGA, B: PIGL, C: GWT1. UniProt ID is indicated between parenthesis for each protein.
